# Efficacy of a Blended Low-Intensity Internet-Delivered Psychological Program in Patients With Multimorbidity in Primary Care: Randomized Controlled Trial

**DOI:** 10.2196/56203

**Published:** 2025-02-10

**Authors:** Alicia Monreal-Bartolomé, Adoración Castro, M Ángeles Pérez-Ara, Margalida Gili, Fermín Mayoral, María Magdalena Hurtado, Esperanza Varela Moreno, Cristina Botella, Azucena García-Palacios, Rosa M Baños, Yolanda López-Del-Hoyo, Javier García-Campayo, Jesus Montero-Marin

**Affiliations:** 1 Research Network on Chronicity, Primary Care and Health Promotion RD21/0016/0005 (RICAPPS), Carlos III Health Institute Madrid Spain; 2 Department of Psychology and Sociology, University of Zaragoza Zaragoza Spain; 3 Aragon Institute for Health Research, IIS Aragon Zaragoza Spain; 4 Research Institute of Health Sciences (IUNICS), University of the Balearic Islands (UIB) Palma de Mallorca Spain; 5 Health Research Institute of the Balearic Islands (IdISBa) Son Espases University Hospital, Building S Palma de Mallorca Spain; 6 Department of Psychology University of the Balearic Islands (UIB) Palma de Mallorca Spain; 7 Mental Health Department, University Regional Hospital of Malaga Málaga Spain; 8 Biomedical Research Institute of Málaga, IBIMA Málaga Spain; 9 Research and Innovation Unit (RD21/0016/0015), Costa del Sol University Hospital, Marbella Málaga Spain; 10 CIBER Physiopathology Obesity and Nutrition (CIBERobn) Carlos III Health Institute Madrid Spain; 11 Department of Clinical and Basic Psychology and Biopsychology, Faculty of Health Sciences, University Jaume I Castellon Spain; 12 Department of Psychological, Personality, Evaluation and Treatment, University of Valencia Valencia Spain; 13 Teaching, Research & Innovation Unit Sant Joan de Déu Health Park Sant Boi de Llobregat Spain; 14 Department of Psychiatry Warneford Hospital University of Oxford Oxford United Kingdom; 15 Consortium for Biomedical Research in Epidemiology & Public Health (CIBERESP) Madrid Spain

**Keywords:** multimorbidity, depression, type 2 diabetes, low back pain, primary care, blended, internet, randomized controlled trial, RCT

## Abstract

**Background:**

Multimorbidity is a highly prevalent phenomenon whose presence causes a profound physical, psychological, and economic impact. It hinders help seeking, diagnosis, quality of care, and adherence to treatment, and it poses a significant dilemma for present-day health care systems.

**Objective:**

This study aimed to assess the effectiveness of improved treatment as usual (iTAU) combined with a blended low-intensity psychological intervention delivered using information and communication technologies for the treatment of multimorbidity (depression and type 2 diabetes or low back pain) in primary care settings.

**Methods:**

A 2-armed, parallel-group, superiority randomized controlled trial was designed for this study. Participants diagnosed with depression and either type 2 diabetes or low back pain (n=183) were randomized to “intervention + iTAU” (combining a face-to-face intervention with a supporting web-based program) or “iTAU” alone. The main outcome consisted of a standardized composite score to consider (1) severity of depressive symptoms and (2a) diabetes control or (2b) pain intensity and physical disability 3 months after the end of treatment as the primary end point. Differences between the groups were estimated using mixed effects linear regression models, and mediation evaluations were conducted using path analyses to evaluate the potential mechanistic role of positive and negative affectivity and openness to the future.

**Results:**

At 3-month follow-up, the intervention + iTAU group (vs iTAU) exhibited greater reductions in composite multimorbidity score (B=–0.34, 95% CI –0.64 to –0.04; Hedges *g*=0.39) as well as in depression and negative affect and improvements in perceived health, positive affect, and openness to the future. Similar positive effects were observed after the intervention, including improvements in physical disability. No significant differences were found in glycosylated hemoglobin, pain intensity, or disability at 3-month follow-up (*P*=.60; *P*=.79; and *P*=.43, respectively). Path analyses revealed that the intervention had a significant impact on the primary outcome, mediated by both positive and negative affect (positive affect: indirect effect=–0.15, bootstrapped 95% CI –0.28 to –0.03; negative affect: indirect effect=–0.14, bootstrapped 95% CI –0.28 to –0.02).

**Conclusions:**

This study supports the efficacy of a low-intensity psychological intervention applied in a blended format on multimorbidity in primary care. It justifies the exploration of the conceptualization of depression in type 2 diabetes as well as the analysis of the implementation of such interventions in routine clinical practice.

**Trial Registration:**

ClinicalTrials.gov NCT03426709; https://clinicaltrials.gov/study/NCT03426709

**International Registered Report Identifier (IRRID):**

RR2-10.1186/S12888-019-2037-3

## Introduction

### Background

Multimorbidity (ie, the presence of ≥2 chronic medical conditions) is a highly prevalent phenomenon [1-4] that affects 1 in 3 adults [5] and has been increasing in recent decades [6-9]. Its presence has a significant physical, psychological, and economic impact and hinders help seeking, diagnosis, the quality of care received, and adherence to treatment [10-14]. Multimorbidity studies conducted in Spain confirm that mental illnesses, particularly major depression, negatively impact quality of life and disability [15,16]. Comorbidity between depression and chronic medical conditions is one of the leading global public health priorities [17]. Although hypertension is very prevalent [15], the most disabling chronic medical conditions are osteoarticular diseases (especially chronic pain), diabetes, and cerebral infarction [15,16]. This study focuses on 2 physical conditions that are comorbid with depression and that involve the greatest disability, loss in quality of life, and higher health care costs: diabetes and chronic pain.

Despite the high prevalence of multimorbidity worldwide, with its consequent demand for care and important health and economic consequences [18], much work remains to be done. Multimorbidity interventions pose a challenge for present-day health care systems [19]. As pointed out by a previous meta-analysis, it is difficult to improve outcomes in people with multimorbidity, although interventions oriented toward depression or specific difficulties and risk factors are promising [17]. There is consensus on the need for a comprehensive assessment to identify patients with multimorbidity who are at risk for negative health outcomes and to simultaneously treat mental and physical comorbidities to prevent functional limitations and future deterioration [20-24]. A comprehensive approach is recommended, addressing not only the medical conditions but also the social, cognitive, and functional issues faced by these patients, as well as a stepped and personalized approach, with therapeutic goals being collaboratively negotiated and regularly re-evaluated throughout the process, for example, by applying the Ariadne principles [22,25]. Patient-oriented approaches, interventions to support self-management, and training for health care professionals appear to be the most frequent elements of interventions with the potential to have a positive impact on patients with multimorbidity [26].

Interventions involving the use of information and communication technologies (ICTs) have been suggested as a promising resource for the provision of adequate and timely support for the self-management of multimorbidity [27-29]. Several studies have demonstrated the effectiveness of personalized, ICT-based interventions for treating depression [30]. However, their effectiveness and cost-effectiveness have not been assessed within a multimorbidity framework. We use the term multimorbidity not only to refer to a specific population of patients but also to the way they are approached and treated [31-33]. Studies focusing on comorbidity deal with only one priority condition over another, instead of addressing multimorbidity [34,35]. As a result, unlike studies using a multimorbidity approach, such as this study, they neglect the bidirectional relationship between the different conditions present and their role or influence on the course of the total index disease. Such approaches, such as using comorbidity, contradict the general recommendation of managing all of a patient’s conditions simultaneously to prevent functional limitations and subsequent decline. The comorbidity concept is useful in secondary and tertiary care settings, while multimorbidity is more useful in primary care (PC) [33].

### Objectives

In this context, this study aimed to evaluate the effectiveness of a blended low-intensity psychological intervention delivered via ICTs for the treatment of multimorbidity (including depression plus either type 2 diabetes or low back pain) in PC settings. We hypothesized that the improved treatment as usual (iTAU) intervention, enhanced by the delivery of ICT-based, low-intensity psychological therapy, would be more effective for ameliorating multimorbidity symptoms in PC compared to a group receiving only iTAU at 3 months after completion of the program.

## Methods

### Study Design

This was a parallel-group, superiority randomized controlled trial (RCT) in which patients receiving treatment as usual by their general practitioners (GPs) were randomized to receive either (1) iTAU or (2) the same iTAU combined with a blended low-intensity internet-delivered psychological intervention, which comprised 2 individual face-to-face sessions and 6 individual web-based therapeutic modules. Upon implementation of the protocol and commencement of the RCT, and ultimately due to the outbreak of the COVID-19 pandemic, the RCT management group was compelled to make several changes to the original study protocol [36]. Three major changes were made to the original protocol [36], all of which were discussed and agreed upon by the trial management group before their implementation and were approved by the Research Ethics Committee of the Autonomous Community of Aragon (CEICA; PI16/0259). Changes were made to (1) the number of participants recruited, (2) the time point measurements, and (3) the mechanistic outcome measures. These changes are addressed in the corresponding sections.

### Participants and Procedure

Participants were recruited at PC health centers of the 3 Spanish autonomous communities participating in this study (Andalusia, Aragon, and the Balearic Islands). The inclusion criteria were as follows: (1) minimum age of 18 years; (2) *Diagnostic and Statistical Manual of Mental Disorders, Fifth Edition* diagnosis of major depression or persistent depressive disorder, of mild or moderate severity, expressed as a Patient Health Questionnaire-9 (PHQ-9) score <14; (3) duration of depressive symptoms of ≥2 months; (4) diagnosis of (a) type 2 diabetes (diagnosis according to criteria of the American Diabetes Association [ADA] [37]) or (b) low back pain (diagnosis of nonspecific chronic low back pain according to the definition established by the European Cooperation in Science and Technology B-13 clinical practice guidelines [38] with a duration of at least 6 months); (5) possession of and ability to use a computer, an internet connection, and a mobile phone; (6) ability to understand oral and written Spanish; and (7) willingness to participate in the study and signing the corresponding informed consent form. The following exclusion criteria were applied: (1) any diagnosis of a disease that might affect the central nervous system (eg, brain condition, traumatic brain injury, or dementia); (2) other psychiatric diagnoses or acute mental illness (eg, substance dependence or abuse, history of schizophrenia or other psychotic disorders, or eating disorders), except for anxiety disorder or personality disorders; (3) any medical, infectious, or degenerative disease that might affect mood; (4) presence of delusional ideas or hallucinations, whether consistent with mood; and (5) suicide risk.

The protocol [36] initially stated that this study would require 63 participants in each arm to detect an expected medium effect size on multimorbidity symptoms [39,40]. Nevertheless, to provide enough statistical power to compare the participants receiving psychological therapy versus those only receiving iTAU, considering the comorbid disease that manifested in conjunction with depression (ie, type 2 diabetes or low back pain), 4 possible subgroups were secondarily established, resulting in a total of 252 participants. In addition, an experimental mortality of approximately 15% was expected [41], which meant that the required sample size was initially estimated at approximately 300 participants. However, challenges arose during the trial setup process in recruiting patients with type 2 diabetes. Specifically, most patients with type 2 diabetes were older adults who lacked access to the internet or email and did not possess the basic computer skills needed to participate in the study. This considerably slowed down the recruitment phase of the trial. The focus on type 2 diabetes was originally decided because it is the most common type of diabetes (approximately 95%) and has a more prevalent association with major depressive disorder, and it has been more widely studied than other types of diabetes [42,43]. However, given that this type of diabetes normally develops at a more advanced age and its risk increases with age, patients with type 2 diabetes were much less inclined to use ICTs and thus showed greater reluctance to participate in this study. After serious consideration, it was decided that the secondary comparison by comorbid disease subgroup would be omitted. A new sample size calculation was then performed that considered each trial arm as a whole entity.

For this purpose, we retained the possibility of detecting a similar intermediate effect size on multimorbidity symptoms to test whether the trend in changes differed between the intervention and control groups. This criterion was considered clinically important in previous research [39,40]. We maintained a statistical power (1–β) of 0.80, a significance level of 5%, and a 1:1 allocation ratio. However, due to the previously mentioned recruitment challenges, we assumed a 1:2 ratio between participants with depression and type 2 diabetes compared to those with depression and low back pain in each group. On the basis of the “time×group” interaction in a general linear repeated measures (RMs) design with Greenhouse-Geisser correction [44], and considering a correlation that decreases monotonically with the time gap between RMs, a base correlation of 0.5, and a decay rate of 0.3, along with the expected 15% mortality, we estimated that a total of 180 participants would be needed. This includes 90 participants in each group, with approximately 30 patients with comorbid depression and type 2 diabetes and 60 patients with comorbid depression and low back pain.

Patients were recruited by GPs working in PC centers of the previously mentioned Spanish autonomous communities, who subsequently sent the referral and consent forms of potential participants to the evaluating investigator. The evaluating investigator then contacted the participants to schedule the screening assessments and recorded the psychological and biological variables to determine their inclusion. Randomization was performed in blocks of patients based on the PC center and comorbid disease (ie, type 2 diabetes or low back pain). An independent researcher unrelated to the research team generated the individual randomization list using a randomization software. A researcher from the research team not involved in any other project-related task, together with an independent GP, performed data monitoring tasks. For further information regarding masking and procedures, refer to the protocol by Monreal-Bartolomé et al [36]. Participants gave their consent for inclusion before learning which treatment they were assigned to and were allowed to withdraw from the study at any time.

### Interventions

#### iTAU at PC Level

All the patients included in this study (both those in the control group and those included in the intervention group) were given their usual treatment by their GPs in PC. This treatment is described as improved because the participating GPs received a training program based on the widely used Spanish guidelines for the treatment of depression in PC, which are based on the National Institute of Health and Care Excellence guidelines [45].

#### Blended Low-Intensity Internet-Delivered Psychological Program

The blended, low-intensity, internet-delivered psychological program was received only by the intervention group. It consisted of 2 face-to-face individual sessions and 6 web-based individual and interactive therapeutic modules. The web-based therapeutic modules were oriented to work on different psychological techniques and therapeutic strategies that have demonstrated their efficacy for treating depression, diabetes, and chronic low back pain, including motivational techniques, psychoeducation on depression and healthy lifestyle, behavioral activation, positive psychology, and mindfulness-based components [46-55]. These modules were supported by multimedia materials (eg, videos and audios) and had an approximate duration of 60 minutes each.

The content of the program is summarized in Tables 1 and 2. The structure of the modules consistently followed the same pattern [36] and concluded with suggested assignments to enable the material covered to be practiced. Before the commencement of each module, the participants were prompted to confirm their completion of the recommended assignments, and they received a response either congratulating them for finishing the tasks or encouraging them to do so. Completing these assignments is considered crucial for consolidating the knowledge acquired in the program and for translating the strategies of the program into skills. To improve engagement, after a period of inactivity on the software (which was scheduled according to the preferences of the patient), users received an email reminder to continue completing the modules. The program was designed to last between 8 and 12 weeks.

**Table 1:** Overview of the program modules.

Module or session	Interventions	Aims and contents
Face-to-face session 1 + M0^a^ (program presentation)	Motivational techniques	To increase participant adherence to face-to-face sessions and assignments. To present the web-based program and train the patients in the procedure and to log in and use it on their home computers.
M1: understanding emotional issues in medical illnesses	Psychoeducation on depression	To develop a new attitude, understanding problems and difficulties as something inherent to daily life, and seeing them as opportunities for learning and growth. The impact of depression on the quality of life and functional capacity of patients as well as on the prognosis of chronic diseases (such as diabetes and chronic pain) was described. In addition, specific techniques and useful and practical tips to reduce stress in daily life were explained.
M2: healthy lifestyle habits and diabetes or chronic pain	Education, information, practical exercises, and specific techniques on how to build and maintain a healthy lifestyle	To work on the healthy lifestyle component, including physical activity, diet, good sleep (relating it to the physical conditions they experience, such as diabetes and chronic pain), and the development of a social support network.
M3: learning how to live	Behavioral activation	To learn to establish and maintain an appropriate level of activity and involvement with life and to schedule activities (especially meaningful ones) and incorporate them into their routine. It is explained that the abandonment of activities that occurs when there is discomfort is not beneficial, but rather worsens the problems. The program also emphasizes the role of activity in mood regulation and physical well-being, highlighting the importance of staying active and engaging in activities that are meaningful, satisfying, and aligned with their values.
M4: life satisfaction	Positive psychology	To recognize the importance of positive emotions and learn strategies that create positive experiences, encouraging participation in enjoyable and meaningful activities, social interaction, improving mood, and supporting effective management of diabetes and chronic pain.
M5: mindfulness and self-compassion	Components of MBCT^b^ and some basic elements of self-compassion	To establish a regular formal practice of mindfulness and self-compassion as well as a regular informal practice. This module includes components of MBCT and MBSR^c^, which have shown positive and promising results in patients with depression, but also diabetes, chronic pain, and multimorbidity [54,56,57]. The section on distancing thoughts to reduce discomfort is particularly important as it allows patients to differentiate between primary and secondary diseases and the causes of each.
Face-to-face session 2	Review of the modules already completed and practice	This semistructured session includes the following objectives: (1) resolution of doubts; (2) performing some of the most important practices, also considering the preferences of each patient (3) emphasis on the continuous practice of the strategies learned; and (4) farewell and completion of the intervention.
M6: So, what happens next?	Relapse prevention and maintenance	To reinforce the strategies learned during the program, encourage participants to continue practicing these strategies throughout the follow-up period and teach them how to identify and cope with future high-risk situations related to depression, diabetes, or chronic pain.

^a^M1, M2, M3, etc, refers to module numbers.

^b^MBCT: mindfulness-based cognitive therapy.

^c^MBSR: mindfulness-bases stress reduction.

**Table 2:** Overview of the program transversal tools.

Transversal tools	Aims and contents
Behavioral activation diary	This tool was designed to focus the attention of the patients to the activities they perform daily; what they spend their time doing; and how this influences their mood, medical condition, and coping ability.
Calendar	This tool provides patients with written information regarding their progress throughout the program.
How am I	This is a tool that provides patients with visual feedback regarding their evolution throughout the program in terms of both their activity level and the intensity of pain as well as their positive (excited, energetic, vital, etc) and negative (upset, fearful, stressed, grumpy, tense, etc) emotionality.

### Measures

#### Overview

During this study, a modification was made to the time point measurements. The initial protocol originally stated [36] that 4 time points would be taken (ie, baseline, posttreatment, 3-month follow-up, and 6-month follow-up), with the last measurement being the primary end point. However, the outbreak of the COVID-19 pandemic in 2020 severely affected life in Spain. As a nationwide lockdown was declared in March 2020, this resulted in PC centers being closed to the public. The COVID-19 pandemic impacted Spanish PC centers, health care professionals, patients, and the general population in various ways—emotionally, socially, and ultimately, in terms of the health care services provided and received. With regard to this RCT, the COVID-19 pandemic outbreak coincided with the final phase of the study, immediately before the last follow-up measurement. Consequently, we had completed the initial baseline measurement as well as delivery of the intervention, postintervention measurement, and the 3-month follow-up measurement, with the 6-month follow-up measurement yet to be conducted. Thus, the most significant impact of the COVID-19 pandemic was on data collection for the primary outcome at 6 months. Several possibilities were considered, although they all pointed to the measurements being seriously compromised in terms of data collection and quality (eg, uncertainty about when PC centers might reopen and social distancing issues). As a result, the feasibility, validity, and accuracy of the estimates—along with the ability to generalize the study results beyond the specific circumstances faced by the Spanish population during the COVID-19 pandemic—were called into question. Therefore, it was decided that the primary end point would be changed from 6-month follow-up to 3-month follow-up after treatment, which was a measure that remained unaffected by the complex and unusual circumstances brought about by the COVID-19 pandemic. Ultimately, the difficult situation at the time prevented us from performing the 6-month follow-up measurement.

#### Sociodemographic Data

The following sociodemographic variables were collected at baseline: self-identified sex, age, marital status, education, employment, and income.

#### Main Outcome

In line with previous RCTs [58-60], the main outcome consisted of a composite score that considered (1) severity of depressive symptoms and (2a) diabetes control or (2b) pain intensity and physical disability.

The severity of depressive symptoms was measured using the PHQ-9 [61,62]. The PHQ-9 consists of 9 questions that correspond to the criteria for diagnosing major depressive disorder in the *Diagnostic and Statistical Manual of Mental Disorders, Fifth Edition*. Respondents rate the frequency of their experiences over the past 2 weeks, ranging from “not at all” to “nearly every day.” The PHQ-9 covers different aspects of depression, including mood, energy levels, sleep, appetite, and concentration. Scores for each question are added up to provide an overall severity score (range 0-27). These scores are categorized according to the severity of depression: minimal or mild depressive symptoms (0-10), mild (10-14), moderate (15-19), and severe (20-27) [63]. The Spanish version of the PHQ-9 has demonstrated adequate psychometric properties [61,62].

Diabetes control was measured using glycated hemoglobin (HbA_1c_). HbA_1c_ is a vital tool for effective diabetes management. It represents the proportion of hemoglobin in the blood that has bound to glucose over an extended period, typically 2 to 3 months. Regular measurement of HbA_1c_ allows tracking progress over time and evaluating whether lifestyle changes or therapy are having an effect. The ADA has defined the following 3 cutoff points: ≤5.6% (nondiabetes), between 5.7% and 6.4% (prediabetes), and ≥6.5% (compatible with a diagnosis of diabetes) [64]. Likewise, the ADA maintains a level of HbA_1c_ ≤7% as the goal for the treatment of patients with diabetes.

Pain intensity and physical disability were assessed using the Faces Pain Scale-Revised (FPS-R) [65] and the Roland-Morris Disability Questionnaire (RMDQ) [66], respectively. The FPS-R is a self-report visual measure in which the patient chooses the face that best represents the level of pain being experienced, with the allocation of a score of 0, 2, 4, 6, 8, or 10, where 0=“no pain” and 10=“very much pain.” The RMDQ was designed to reliably determine the level of physical disability (ie, the limitation in the performance of daily activities) resulting from nonspecific low back pain. It ranges between 0 (absence of disability for back pain) and 24 (maximum possible disability). A change in the score on this scale is clinically significant if it is ≥2 [66-68]. The Spanish version of the RMDQ has demonstrated adequate psychometric properties [66].

The composite score was obtained by combining these components after weights were assigned to them. For (2a), weightage of 50% was assigned both to the severity of depressive symptoms and diabetes control. For (2b), first, the same weightage of 50% was assigned to pain intensity and physical disability (to obtain a composite, including both), and, second, to the severity of depressive symptoms and pain (which included the previous pain measure of intensity and physical disability). These equal weights were added together to yield a standardized composite score that reflected the combination of the 3 components mentioned earlier. This composite score provides a comprehensive measure of comorbidity that considers both depressive symptoms and either diabetes control or pain intensity and physical disability in a single metric.

#### Secondary and Mechanistic Outcomes

Perceived health status was assessed as a secondary outcome using the 12-item Short Form Survey (SF-12) [69]. The SF-12 questionnaire is widely used in both clinical and research settings. A total score is calculated after coding and transforming the items that range from 0 (worst possible health status) to 100 (best possible health status) [70]. The SF-12 has demonstrated appropriate psychometric qualities [71,72] and has been validated in Spanish [73].

As a new addition to the protocol, it was decided that a measure of affectivity (ie, positive and negative affect) would be added as a potential mechanistic variable. The rationale for this was to be able to evaluate the potential effects of changes in affectivity on promoting better outcomes. For this purpose, the Positive and Negative Affect Schedule (PANAS) was used [74,75]. This self-report measure consists of 2 subscales: positive and negative affect. Each of the subscales includes 10 adjectives, which must be rated on a scale ranging from 1=“not at all or very slightly” to 5=“very much,” depending on the degree to which each adjective describes the state of mind in which participants generally find themselves. The maximum score is 50 for each of the subscales. This scale has shown good psychometric properties [74-76].

Finally, we used the Openness to the Future Scale (OFS) [77] to specifically measure positive affectivity toward the future as a potential mechanism. This variable can be an indicator of psychological adjustment and a protective factor for mental health. The OFS is a self-reported questionnaire composed of 10 items that are scored on a scale from 1 (“strongly disagree”) to 5 (“strongly agree”). A total openness to the future score is obtained by summing the scores of all items (after reversing item 6). The OFS has shown good psychometric properties in the Spanish population [77].

### Data Analyses

Data were analyzed using Stata V.18.0 statistical software [78]. First, sociodemographic and outcome descriptive data at baseline (ie, preintervention) were analyzed using frequencies (percentages), medians (IQR), and means (SD), according to their level of measurement and statistical distribution. We evaluated the success of randomization by visual inspection.

The primary analysis consisted of a comparison between the intervention + iTAU and iTAU groups at a 3-month follow-up after the intervention ended, considering the main outcome as a continuous variable. The main outcome was a composite score [36] that included the following: (1) depressive symptom severity using the PHQ-9 [61] and (2a) control of diabetes measured by HbA_1c_ or (3b) pain intensity using the FPS-R [65] and physical disability using the RMDQ [66]. The composite score was weighted to give a continuous standardized aggregate score. The primary analysis was performed using an RM design on a modified intention-to-treat (ITT) basis, that is, we analyzed complete cases due to the high proportion of missingness and explored patterns of missing data. We used multilevel linear mixed regression models with the restricted maximum likelihood method for the estimation of parameters, controlling for age and sex as covariates. The “treatment-by-time” interaction was calculated to determine possible differences between the study arms. The slope coefficient (B), representing the between-group adjusted mean difference change (ie, the interaction term), and its 95% CI were calculated (within-group adjusted mean difference changes and 95% CIs are also provided). Hedges *g*, as an effect size measure of between-group differences, was calculated from the raw data, with Hedges *g*=0.2 (small effect), Hedges *g*=0.5 (intermediate effect), and *g*≥0.8 (large effect) [79]. We used a 2-sided test with a .05 significance level.

The same analytical approach was used to perform secondary analyses for the main outcome at postintervention as well as for the components of the composite score and for the secondary and mechanistic outcomes at postintervention and 3-month follow-up. We corrected for multiple comparisons by adjusting the significance threshold based on the number of comparisons and the rank of the *P* value according to the Benjamini-Hochberg procedure [80].

Additional post hoc sensitivity analyses were also conducted using complier average causal effect (CACE) or instrumental variable (IV) methods [81] to further investigate the impact of compliance with the program on the composite (primary outcome) at postintervention and at 3-month follow-up, while accounting for potential hidden confounding relationships. A participant in the intervention + iTAU arm was considered a complier if they attended the 2 face-to-face sessions and completed the 6 web-based modules. For this purpose, a 2-stage least squares IV approach was used. In the first stage of regression, marital status, employment, and diagnosis were included as predictors of compliance. In the second stage of regression, age, sex, and the composite at preintervention were introduced as predictors of the outcome at postintervention or 3-month follow-up. The allocated group was used as an IV to define compliance. Results are presented in terms of unstandardized regression coefficients, along with their corresponding 95% CI and *P* values.

The role of positive affect, negative affect, and openness to the future as mediators of improvements in the main outcome was explored. For this purpose, (1) the primary outcome at follow-up was considered the dependent variable; (2) pre-post differential scores of positive affect, negative affect, and openness to the future were calculated and included as process variables; and (3) the group condition (with 2 possibilities: intervention + iTAU vs iTAU) was considered the independent variable. Models included the main outcome at preintervention, age, and sex as covariates. The mediating analyses were conducted using path analyses for continuous dependent variables. Standardized regression coefficients for bootstrapped indirect effects were estimated, along with their 95% CIs based on 10,000 bootstrapped samples. A significant mediating effect was considered when the 95% CI did not include zero [82].

### Ethical Considerations

This study was approved by the research ethics committee of each of the autonomous communities involved (CEICA Aragon: PI16/0259, CEI Balearic Islands: IB 3402/17 PI, and the Regional Ethics and Research Committee of the province of Malaga: 03/2017 ICPS 2) and was designed in accordance with the ethical standards laid down in the Declaration of Helsinki and its later amendments. Modifications to the published protocol [36] were approved by the research ethics committee of the autonomous community corresponding to the leading group (CEICA Aragon, PI16/0259). Written informed consent was obtained before screening, and exclusion criteria were applied afterward. As this study involved the use of the internet, AES strategies for data encryption and personal password use were implemented to ensure the protection of personal information. The data were treated anonymously and confidentially and were used solely for the purposes of the study. Study participants did not receive any compensation for their participation, other than receiving improved treatment for their condition.

## Results

### Flow, Baseline Characteristics, and Compliance

**Figure 1:**
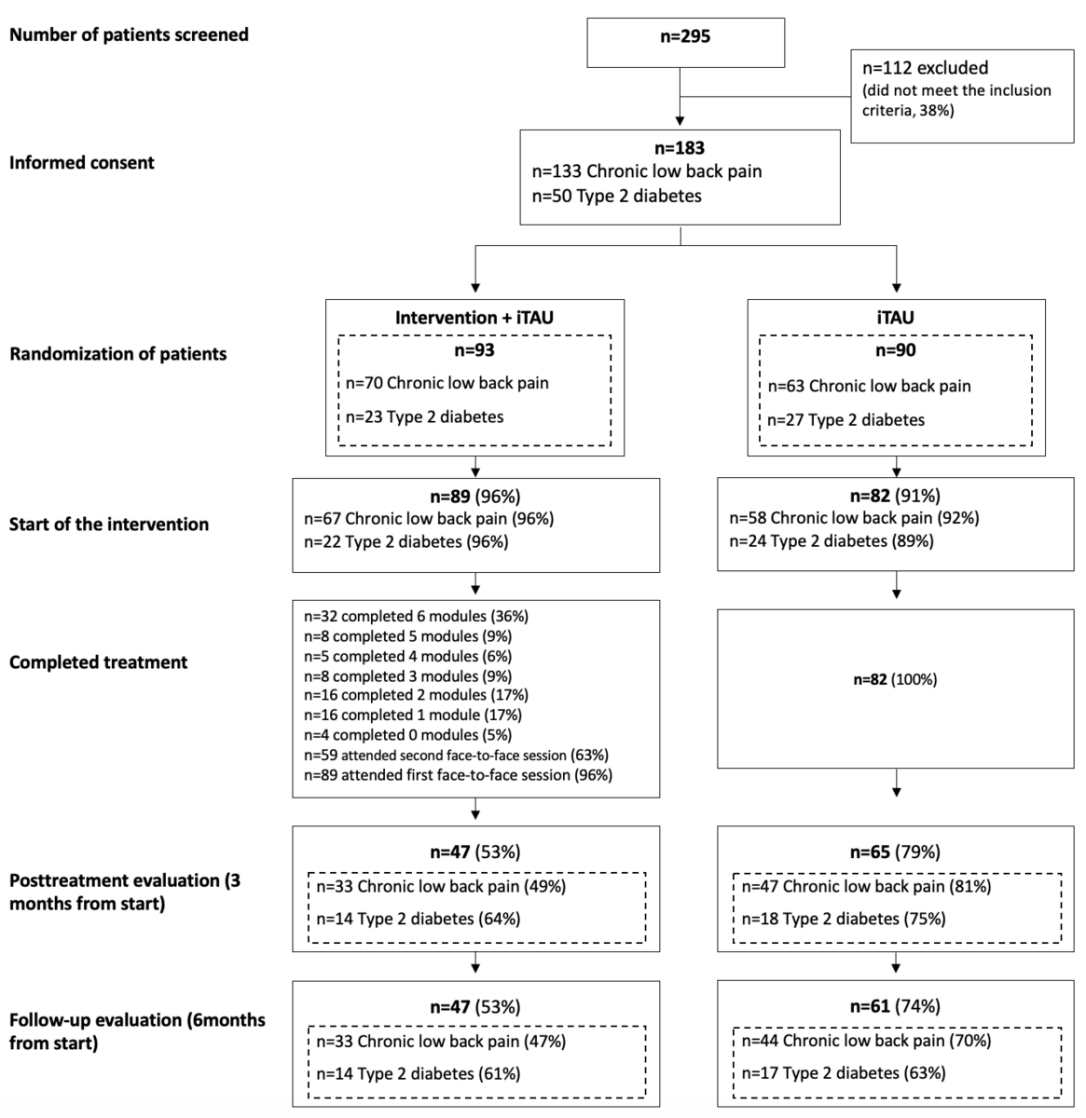
Participant flow diagram. Numbers at posttreatment and follow-up evaluations reflect those cases in which at least 1 of the variables that make up the composite multimorbidity main outcome measure were obtained. iTAU: improved treatment as usual.

As shown in Figure 1, after excluding 112 (37.9%) participants (who did not meet the inclusion criteria) from the initial 295, the remaining 183 (62.1%) individuals were randomly assigned to 1 of the 2 experimental conditions (intervention + iTAU: n=93, 50.8%; iTAU: n=90, 49.2%). The participants were mostly women (132/183, 72.1%) with a mean age of 51.36 (SD 11.3) years, and there were no important differences in either sociodemographic or clinical characteristics between the 2 arms that might suggest prognostic strength (Table 3). In line with the CONSORT (Consolidated Standards of Reporting Trials) guidelines for reporting parallel-group RCTs and following the recommendation to avoid conducting probability tests on potential baseline differences that may have occurred by chance due to random assignment, we did not perform such hypothesis testing. This type of testing is unnecessary because it assesses the likelihood that observed baseline differences happened by chance, and as random assignment has already been performed, this is already understood. Instead, we based our comparisons of sociodemographic and clinical data at baseline on the size of potential chance imbalances and their prognostic relevance [83,84]. In this regard, there were no significant differences in sociodemographic or clinical characteristics between the two groups that could suggest prognostic strength (Table 2). Therefore, no additional covariates were included in the subsequent analytical models beyond those prespecified in the protocol, namely, age and sex.

**Table 3:** Baseline characteristics of patients by treatment group (N=183).

Characteristics			Intervention + iTAU^a^ (n=93)	iTAU alone (n=90)
**Sociodemographic data**				
	Age (y), mean (SD)		51.20 (11.16)	51.51 (11.51)
	Sex (female), n (%)		74 (79)	58 (65)
	**Marital status^b^, n (%)**			
		Married or relationship	56 (61)	54 (60)
		Single	17 (19)	16 (18)
		Separated or divorced	17 (19)	12 (13)
		Widowed	2 (2)	8 (9)
	**Place of residence,** **n (%)**			
		Own home	79 (86)	75 (83)
		Relative’s home	4 (4)	5 (6)
		Neighbor’s or friend’s home	0 (0.0)	1 (1)
		Other	9 (10)	9 (10)
	**Education, n (%)**			
		No studies	0 (0)	6 (7)
		Primary studies	22 (24)	17 (19)
		Secondary studies	55 (60)	39 (43)
		Tertiary studies	15 (16)	28 (31)
	**Employment, n (%)**			
		Unemployed	24 (26)	28 (31)
		Employed	24 (26)	38 (42)
		Home duties	2 (2)	5 (6)
		Student	1 (1)	0 (0)
		Sick leave	22 (24)	8 (9)
		Retired	19 (21)	11 (12)
	**Income^c^, n (%)**			
		≤National minimum wage (US $600)	25 (27)	20 (22)
		1-2 × national minimum wage (US $600)	30 (33)	31 (34)
		2-4 × national minimum wage (US $600)	22 (24)	26 (29)
		>4 × national minimum wage (US $600)	1 (1)	4 (4)
**Clinical data**				
	**Diagnosis, n (%)**			
		Pain	70 (75)	63 (70)
		Diabetes	23 (25)	27 (30)
	**Medications, median (IQR)**		3 (2-4)	3 (2-4)
		Analgesics, n (%)	57 (61)	50 (56)
		Antidepressants, n (%)	39 (42)	37 (41)
		Antiepileptics, n (%)	16 (17)	7 (8)
		Antipsychotics, n (%)	1 (1)	0 (0)
		Antidiabetics, n (%)	21 (23)	23 (26)
		Antithyroid, n (%)	5 (5)	4 (4)
		Anxiolytics, n (%)	45 (48)	33 (37)
		Cardiovascular medication, n (%)	32 (34)	34 (38)
		Corticosteroids, n (%)	4 (4)	4 (4)
		Insulin, n (%)	5 (5)	7 (8)
		NSAIDs^d^, n (%)	1 (1)	0 (0)
		Other, n (%)	30 (32)	36 (40)
	Composite (range –2.25 to 2.25), mean (SD)		–0.02 (0.74)	0.05 (0.81)
	PHQ-9^e^ (range 0-27), mean (SD)		14.48 (5.34)	13.84 (5.79)
	RMDQ^f^ (range 0-24), mean (SD)		13.54 (5.54)	14.38 (6.13)
	FPS-R^g^ (range 0-10), mean (SD)		4.86 (2.15)	5.11 (2.47)
	HbA_1c_^h^ (diabetes: ≥6.5), mean (SD)		6.78 (0.66)	7.77 (2.48)
	SF-12^i^ (range 0-100), mean (SD)		30.33 (19.28)	31.84 (18.36)
	PANAS^j^-positive subscale (range 10-50), mean (SD)		20.32 (6.48)	20.97 (7.34)
	PANAS-negative subscale (range 10-50), mean (SD)		26.35 (7.72)	26.06 (8.50)
	OFS^k^ (range 10-50), mean (SD)		30.82 (8.12)	29.97 (8.06)

^a^iTAU: improved treatment as usual.

^b^Marital status, place of residence, education, and employment: 1 missing in the intervention arm.

^c^Income: 15 missing in the intervention arm, and 9 missing in the control arm.

^d^NSAID: nonsteroidal anti-inflammatory drug.

^e^PHQ-9: Patient Health Questionnaire-9.

^f^RMDQ: Rolland-Morris Disability Questionnaire.

^g^FPS-R: Faces Pain Scale-Revised

^h^HbA_1c_: glycosylated hemoglobin.

^i^SF-12: 12-item Short Form Survey.

^j^PANAS-positive: Positive and Negative Affect Scale.

^k^OFS: Openness to the Future Scale.

On average, the PHQ-9 indicated moderate levels of depression (mean 14.18, SD 5.55) in the total sample, the RMDQ suggested a dysfunctional low back pain status (mean 13.93, SD 5.82) in the subgroup with low back pain, and the HbA_1c_ value confirmed the presence of diabetes (mean 7.09, SD 1.51) in the type 2 diabetes subgroup. More than half of the sample (107/183, 58.5%) was taking analgesics, which was the most common medication. In total, 95% (89/93) of the participants in the intervention + iTAU arm attended the first face-to-face session, and 63% (59/93) of the participants attended the second face-to-face session. The median (IQR) number of modules attended in the intervention arm was 4 (out of 7), with a mean of 4.02 (SD 2.75). Postintervention retention rates in the primary outcome (ie, composite score) were 37% (35/93) in the intervention + iTAU arm and 54% (49/90) in the iTAU arm, with rates of 49% (46/93) and 55% (50/90), respectively, at follow-up. At the 3-month follow-up (primary end point), higher age, being separated or divorced, and having a diabetes diagnosis (as well as taking antidiabetics and insulin) were significantly associated with a higher probability of attrition. On the other hand, being employed, engaging in household chores, being on sick leave, and taking analgesics and antiepileptics were significantly associated with a lower probability of study attrition (Multimedia Appendix 1).

### Effects on Primary and Secondary Outcomes at Primary End Point

The study outcomes are reported by trial arm status at postintervention (Multimedia Appendix 2) and 3-month follow-up (Table 4). At 3-month follow-up (primary end point), the within-group analyses of the intervention + iTAU group based on a modified ITT basis revealed significant improvements in the composite score (main outcome) as well as in depression (PHQ-9), perceived health status (SF-12), positive affect (PANAS-positive), negative affect (PANAS-negative), and openness to the future (OFS; Table 4). However, there were no significant effects on HbA_1c_ or pain intensity and disability (RMDQ and FPS-R). At 3-month follow-up, the within-group analyses of the iTAU group based on a modified ITT basis showed no significant effects in any of the outcomes (Table 4). At 3-month follow-up, there was evidence that the intervention + iTAU group achieved a significantly greater reduction in the composite score (main outcome) compared to iTAU (B=–0.34, 95% CI –0.64 to –0.04), with small-to-medium effects (Hedges *g*=–0.39). Furthermore, compared to iTAU, the intervention + iTAU group showed greater reductions in depression (PHQ-9: B=–3.92, 95% CI –5.70 to –2.15) and negative affect (PANAS-negative: B=–3.67, 95% CI –6.63 to –0.71) and greater improvements in perceived health status (SF-12: B=9.04, 95% CI 3.21-14.87), positive affect (PANAS-positive: B=4.73, 95% CI 2.01-7.45), and openness to the future (OFS: (B=4.73, 95% CI 2.01-7.45), with small-to-large effects (Hedges *g* ranging from 0.36 to 1.13 in absolute value). However, no significant differences in HbA_1c_ or pain intensity and disability (RMDQ and FPS-R) were identified in the comparison between the intervention + iTAU group and the iTAU group at 3-month follow-up.

**Table 4:** Descriptive statistics and main comparisons for primary and secondary outcomes at 3-month follow-up.

				Participants, n (intervention, iTAU^a^)		Intervention + iTAU, mean (SD)		iTAU alone, mean (SD)		Within group, B (95% CI)				Between group				
										Intervention + iTAU		iTAU alone		*d*		*P* value^b^		B (95% CI)
**Primary outcome**																		
	**Composite (range –2.25 to 2.25)**			46, 50														
		Baseline			–0.10 (0.79)		–0.01 (0.80)		—^c^		—		—		—		—	
		Follow-up			–0.21 (0.86)		0.19 (0.86)		–0.13 (–0.37 to – 0.02)		0.20 (–0.02 to 0.43)		–0.39		.02		–0.34 (–0.64 to –0.04)	
**Secondary outcomes**																		
	**PHQ-9^d^ (range 0-27)**			47, 61														
		Baseline			13.89 (5.97)		13.41 (5.45)		—		—		—		—		—	
		Follow-up			9.79 (6.46)		13.05 (6.45)		–4.30 (–5.56 to –3.04)		–0.50 (–1.73 to 0.72)		–0.65		<.001		–3.92 (–5.70 to –2.15)	
	**RMDQ^e^ (range 0-24)**			33, 44														
		Baseline			13.36 (5.49)		14.00 (6.05)		—		—		—		—		—	
		Follow-up			13.06 (6.28)		14.39 (6.18)		–0.36 (–1.90 to 1.18)		0.04 (–0.87 to 0.95)		–0.12		.43		–0.74 (–2.58 to1.10)	
	**FPS-R^f^ (range 0-10)**			33, 44														
		Baseline			4.67 (1.78)		5.00 (2.18)		—		—		—		—		—	
		Follow-up			5.21 (2.50)		5.73 (2.05)		0.43 (–0.37 to 1.23)		0.65 (–0.13 to 1.43)		–0.10		.79		–0.14 (–1.13 to 0.86)	
	**HbA_1c_^g^ (diabetes: ≥6.5)**			16, 5														
		Baseline			6.73 (0.66)		7.54 (1.40)		—		—		—		—		—	
		Follow-up			6.89 (1.31)		7.54 (2.08)		0.23 (–0.26 to 0.72)		–0.01 (–0.78 to 0.77)		0.18		.60		0.23 (–0.61 to 1.07)	
	**SF-12^h^ (range 0-100)**			47, 60														
		Baseline			33.91 (19.43)		33.17 (17.81)		—		—		—		—		—	
		Follow-up			43.46 (24.01)		33.04 (21.95)		10.81 (5.98 to15.64)		1.19 (–2.25 to 4.63)		0.52		.002		9.04 (3.21 to 14.87)	
	**PANAS^i^-positive subscale (range 10-50)**			44, 59														
		Baseline			20.68 (7.14)		21.47 (7.05)		—		—		—		—		—	
		Follow-up			25.36 (9.77)		21.24 (8.88)		4.84 (2.04 to 7.64)		–0.03 (–1.86 to 1.80)		1.13		.001		4.73 (2.01 to 7.45)	
	**PANAS-negative subscale (range 10-50)**			44, 59														
		Baseline			25.89 (8.50)		27.17 (8.46)		—		—		—		—		—	
		Follow-up			21.09 (8.34)		25.47 (9.31)		–5.05 (–7.14 to – 2.95)		–1.32 (–3.14 to 0.50)		–0.36		.02		–3.67 (–6.63 to –0.71)	
	**OFS^j^ (range 10-50)**			47, 60														
		Baseline			31.02 (7.86)		30.43 (8.18)		—		—		—		—		—	
		Follow-up			34.06 (9.01)		30.32 (8.13)		3.13 (0.98 to 5.29)		0.03 (–1.60 to 1.68)		0.39		.02		3.08 (0.55 to 5.62)	

^a^iTAU: improved treatment as usual.

^b^All significant results remained significant after correction for multiple comparisons using the Benjamini-Hochberg method.

^c^Not applicable.

^d^PHQ-9: Patient Health Questionnaire-9.

^e^RMDQ: Rolland-Morris Disability Questionnaire.

^f^FPS-R: Faces Pain Scale-Revised.

^g^HbA_1c_: glycosylated hemoglobin.

^h^SF-12: 12-item Short Form Survey.

^i^PANAS: Positive and Negative Affect Scale.

^j^OFS: Openness to the Future Scale.

### Effects on Primary and Secondary Outcomes at Secondary End Point

At postintervention, the within-group analyses of the intervention + iTAU group based on a modified ITT basis revealed significant improvements in the composite score (main outcome) as well as in depression (PHQ-9), pain intensity (RMDQ), HbA_1c_, perceived health status (SF-12), positive affect (PANAS-positive), negative affect (PANAS-negative), and openness to the future (OFS); however, there were no significant effects on pain disability (FPS-R; Multimedia Appendix 2). At postintervention, the within-group analyses of the iTAU group based on a modified ITT basis showed a significant worsening in the composite score (main outcome); however, there were no other significant effects (Multimedia Appendix 2). At postintervention, there was evidence that the intervention + iTAU group achieved a significantly greater reduction in the composite score (main outcome) compared to iTAU (B=–0.63, 95% CI –0.94 to –0.31), with large effects (Hedges *g*=–0.85). Furthermore, the intervention + iTAU group also showed greater reductions in depression (PHQ-9: B=–5.02, 95% CI –6.77 to –3.26), low back pain disability (RMDQ: B=–2.28, 95% CI –4.10 to –0.46), and negative affect (PANAS-negative: B=–5.52, 95% CI –8.45 to –2.60) and greater improvements in perceived health status (SF-12: B=13.55, 95% CI 7.77-19.33), positive affect (PANAS-positive: B=5.25, 95% CI 2.56-7.93), and openness to the future (OFS: B=3.16, 95% CI 0.64-5.67), with small-to-large effects (Hedges *g* ranging from 0.38 to 0.88 in absolute value). However, no significant differences in HbA_1c_ and pain intensity (FPS-R) were identified in the comparison between the intervention + iTAU group and the iTAU group at postintervention.

### Effect of Compliance With the Program

The results of the CACE or IV analyses represent an estimation of the intervention effect among the subpopulation of compliers in the intervention arm, compared to those in the control arm who would have complied with the intervention had they been offered it. The CACE or IV analyses indicated evidence of a relationship between the completion of the program and the composite score (main outcome) at postintervention (B=–1.04, 95% CI –1.56 to –0.52; *P*<.001). These analyses also showed evidence of a relationship between the completion of the program and the composite (main outcome) at 3-month follow-up (B=–0.54, 95% CI –1.01 to –0.07; *P*=.03).

### Analysis of Mediating Variables

The results of the path analyses on the primary outcome (composite score) are detailed in Table 5, where the independent variable is the group condition (intervention + iTAU vs iTAU). Three models controlled for the main outcome at baseline, age, and gender were tested (ie, positive affect, negative affect, and openness to the future as potential mediators of the effect of the intervention on the outcome); however, only positive affect and negative affect showed a significant indirect effect (positive affect: ab=–0.15, bootstrapped 95% CI –0.28 to –0.03 and negative affect: ab=–0.14, bootstrapped 95% CI –0.28 to –0.02). After controlling for the mediators, the direct effect (path c) of the intervention was significant and of the same sign as the indirect effects in both models, suggesting a “complementary mediation.”

**Table 5:** Direct and bootstrapped indirect effects in the mediational models.

Main outcome^a^ and mediators^b^ (*R^2^*)^c^, and direct effects						Indirect effects				
		Path^d^	Coefficient^e^	*P* value^f^	Path		Coefficient^g^		95% CI^h^	
**Composite (0.57)**										
	***Positive affect*** **(0.16)**					a × b		–0.15		–0.28 to –0.03
		a^i^	0.76	.001						
		b^j^	–0.19	.03						
		c′^k^	–0.57	.001						
		c^l^	–0.42	.01						
***Composite*** **(0.55)**										
	***Negative affect*** **(0.15)**					a × b		–0.14		–0.28 to –0.02
		a	–0.60	.007						
		b	0.22	.007						
		c′	–0.56	.001						
		c	–0.42	.009						
***Composite*** **(0.58)**										
	***Openness to the future*** **(0.12)**					a × b		–0.06		–0.18 to 0.01
		a	–0.15	.07						
		b	0.37	.10						
		c′	–0.54	.001						
		c	–0.48	.003						

^a^The dependent variable (main outcome) is the composite score at 3-month follow-up.

^b^The potential mediators, highlighted in italics (positive affect, negative affect, and openness to the future), were based on pre-post change scores.

^c^*R*^2^: variance explained by regression models.

^d^Path coefficients are (standardized) ordinary least squares–based regression coefficients.

^e^Coefficient: (standardized) slope.

^f^*P* value related to *t* test.

^g^The product of “ab” is the bootstrapped indirect effect using 10,000 samples.

^h^It is the 95% CI of the bootstrapped indirect effect using 10,000 samples.

^i^a: the direct path between the independent variable and the mediator.

^j^b: the direct path between the mediator and the outcome.

^k^c′: total effects.

^l^c: direct effect of the independent variable on the dependent variable after adjustment for mediating effects.

## Discussion

### Principal Findings

The main aim of this trial was to evaluate the effectiveness of a low-intensity psychotherapy program (intervention + iTAU) applied in a hybrid form with face-to-face and internet-based sessions (ie, blended), for the treatment of multimorbidity between mild to moderate severity depression and either type 2 diabetes or chronic low back pain in PC, compared to a group that only received iTAU. It was observed that the intervention + iTAU group achieved a significantly greater reduction in the composite score (main outcome) compared to iTAU, with large effects at postintervention and small-to-medium effects at follow-up. In addition, compared to iTAU, the intervention + iTAU group showed greater reductions in depressive symptomatology, low back pain disability, and negative affect at postintervention, although not at follow-up, where only reductions in depression and negative affect were maintained. On the other hand, greater improvements in perceived health, positive affect, and openness to the future were observed in the intervention + iTAU group versus iTAU, with small-to-large effects at both time points. However, no significant differences in HbA_1c_ or pain intensity were identified in the comparison between the intervention + iTAU group and the iTAU, either at postintervention or 3-month follow-up.

### Comparison to Prior Work

These results align with previous literature, indicating the effectiveness of psychological intervention programs for multimorbidity in improving depressive symptomatology, whether delivered face-to-face or through ICTs [20,35,49,51,52,85-95]. With regard to pain, the few studies that tested psychotherapy tools delivered via ICTs in these patients showed varying results [96]. While some studies achieved improvements in either disability and pain intensity, or both [92,97-99], others achieved no or only partial improvement in these variables [93,100-103]. Differences in the results may be attributed to the significant heterogeneity among these studies, such as variations in the type of intervention, the presence of pain comorbidities, the measurement instruments used, and the existence of comorbid depression. Among the studies that specifically had chronic low back pain and depression as inclusion criteria and were conducted entirely through ICTs, improvements in pain intensity or associated disability were not observed 6 months after randomization [99,102]. However, one of them did show improvements postintervention [99]. It is possible that pain intensity, being a variable that is difficult to modify, exhibits effects that are lost earlier, and this was evident in both cases, although the loss occurred in the medium term. As far as we know, there are no studies applying blended psychotherapy in patients with chronic pain and depression. Therefore, reaching a solid conclusion in this regard is challenging, highlighting the need for further studies in this field.

Our results present a contrast with findings from other studies that used psychological interventions in patients with diabetes and depression [57,104-109]. In those studies, both cognitive behavioral therapy and interventions incorporating mindfulness and self-compassion components appeared effective in glycemic control. However, it is worth noting that these interventions were primarily delivered in a face-to-face design. In studies where interventions were applied via the internet or telephone, improvements in glycemic control were not achieved [35,90,91,94,110-113]. Thus, the method of implementing the intervention could play a decisive role. Given that our intervention was blended, such effects might have been mitigated. This could also be associated with the small sample size (resulting in reduced statistical power) achieved when recruiting patients with diabetes, along with the limited follow-up time (3 months). The HbA_1c_ variable represents the proportion of hemoglobin in the blood that has bound to glucose over an extended period. Therefore, a more extended follow-up time may be necessary to observe significant results, as demonstrated by Hoyo et al [114], where HbA_1c_ levels continued to decrease at 12- and 18-month follow-ups.

With regard to the observed improvements in perceived health, the use of a blended model rather than a solely web-based approach may have led these improvements to stand out, particularly in comparison to other nonblended studies [90,110]. In those studies, only some of the dimensions of perceived health showed improvement, or improvements were not achieved at the intragroup level. A study that applied a face-to-face mindfulness-based cognitive therapy (MBCT) intervention in patients with diabetes and low levels of emotional well-being found that MBCT was more effective for improving perceived health than the waitlist control group [115]. Conversely, in the case of pain, a previous face-to-face delivered study that compared cognitive behavioral therapy, mindfulness-based stress reduction, and a waiting list showed improvements in perceived mental health only [116].

In the case of improvements in negative and positive affect, a situation similar to that observed for the previous variables arose, with varied results across different studies [93,117,118] Those studies exhibit considerable heterogeneity, differing in crucial characteristics such as the type of intervention, delivery method (eg, face-to-face, telephone, and web-based vs mixed), and the nature of the medical conditions. This heterogeneity makes it challenging to compare them with each other and with this study. Our results underscore the crucial role of affect, both positive and negative, as potential mediators in the functioning of the intervention, influencing improvements in the composite main outcome. Understanding how affective states mediate the impact of the intervention is pivotal for tailoring and optimizing future treatment developments. This finding emphasizes the need for interventions that consider the affective dimension, not only addressing the negative valence of depressive symptoms but also enhancing positive emotional experiences. While this study contributes to this understanding, it is noteworthy that similar results were found when using MBCT to reduce the risk of relapse or recurrence in major depressive disorder [119].

Finally, improvements in openness to the future could not be compared with any previous studies due to the novelty of the construct [77]. It is interesting to understand the positive future expectations of patients both before and after receiving an intervention for any health problem. Specifically, the measure used in this study incorporates different aspects, such as the positive illusion of control, the active process of accepting future scenarios, and the confidence and commitment in one’s ability to plan for desired outcomes and to cope with adversity. Nevertheless, this variable did not show any potential mediating effects on the main composite score.

### Strengths and Limitations

This is the first study to use blended models involving ICTs in the treatment of multimorbidity. It focused on a particularly prevalent, disabling, and challenging condition in clinical practice, multimorbidity between depression and type 2 diabetes or chronic low back pain. Furthermore, an evidence-based design and intervention were proposed, adhering to the recommendations of major clinical guidelines and previous research. The intervention specifically targeted risk factors, such as depression, and addressed functional difficulties. It was centered on patients and their specific needs, offering support for self-management, adopting a comprehensive and personalized approach, and incorporating therapeutic objectives negotiated and reassessed throughout the process in accordance with the principles by Ariadne [22].

However, the study has significant limitations. First, a notable challenge was the high attrition rate, which impacted the statistical power of this study. Recruitment and retention difficulties in RCTs targeting comorbid physical and mental illnesses have been documented in prior studies [120,121], and specific details of this study will be discussed in a forthcoming publication focused on implementation. Nevertheless, it is important to highlight that, in line with the earlier recruitment challenges, dropouts were more prevalent among older patients and those with type 2 diabetes. This trend might be attributed to challenges in managing the use of internet and email and the acquisition of the basic computer skills required for active participation. Similar observations were made in a study by Clarke et al [35] focusing on patients with type 2 diabetes and depression. This study reported higher attrition and mean age compared to other ICT-delivered interventions in chronic low back pain and depression [99,102] and diabetes and depression [90,91]. It is noteworthy that the latter studies also included type 1 diabetes, contributing to differences in the age distribution of participants. There is also a proposal that patients with diabetes and mild depression might perceive low mood as a feature of their diabetes rather than a separate condition to be treated, potentially influencing treatment adherence and completion [122]. As suggested by other authors [35], further exploration of the conceptualization of depression in type 2 diabetes and its impact on program uptake and the benefit of treatment is recommended. Regardless, the low recruitment and retention rate in this study, particularly for older patients with diabetes owing to their lack of basic computer skills, could affect the generalizability of the results. Second, similar to many clinical trials, this study faced disruptions due to the COVID-19 pandemic [123], preventing the implementation of necessary follow-up measures, particularly for patients with diabetes. Given their heightened vulnerability to the virus, this situation led to the loss of valuable follow-up data. Trials that stop earlier for reasons independent of trial findings are unlikely to introduce bias because of their premature termination [124]; however, changing the primary end point from a 6-month follow-up to a 3-month follow-up in response to the challenges posed by the COVID-19 pandemic offers a different time perspective than originally proposed. We lack information on the effect sizes at 6 months, and the true effect size may vary between the 3-month and 6-month time points. In this sense, future research might benefit from the inclusion of longer follow-up periods. Third, we observed a varying proportion of losses between the intervention and control groups postintervention, although this effect disappeared at the subsequent primary end point. Similar effects were noted in prior studies [35,90,91,102,111] and could be attributed to some extent to the control groups being wait-listed, leading participants to initially complete the assessment and refrain from dropping out in anticipation of receiving the intervention at a later point. Although modified ITT and CACE or IV approaches provided useful insights into the impact of treatment on those who completed the study and on those who completed the treatment, we must not overlook this potential attrition bias and the possible limitation when generalizing the results to the entire population, where adherence can be variable. Nevertheless, the fact that the effect of the losses disappears at the primary end point indicates that the initial bias was not significant in the long term. A more comprehensive consideration of these implementation aspects will be presented in a subsequent paper. Finally, we did not measure other diseases besides depression, diabetes, and chronic pain; therefore, we are unaware of how the program might work with other potentially present conditions. Future studies should take into account a wider range of potential disorders among the participating patients.

### Conclusions

This study supports the efficacy of a low-intensity psychological intervention applied in a blended form for multimorbidity in PC. Promising outcomes are particularly noted in the psychological dimension, showing improvements in depression, perceived health, positive and negative affect, and openness to the future. However, the findings in the physical dimension are mixed, indicating variable results in pain and disability reduction, promising results in short-term disability reduction, and no improvements in diabetes control. We encourage further research to validate the findings of this study, including the exploration of subgroups that could not be adequately examined due to the discussed limitations. In addition, we advocate an exploration of the conceptualization of depression in type 2 diabetes to shed light on its effects on adherence indicators, acceptance, and the efficacy of psychological interventions in these patients. Analyzing the implementation of such interventions in routine clinical practice is also warranted.

## Appendix

Multimedia Appendix 1 Variables at baseline as predictors of missingness.

Multimedia Appendix 2 Descriptive statistics and main comparisons at postintervention.

Multimedia Appendix 3 CONSORT-eHEALTH checklist (V 1.6.1).

## Abbreviations

ADA: American Diabetes Association
CACE: complier average causal effect
CEICA: Research Ethics Committee of the Autonomous Community of Aragon
CONSORT: Consolidated Standards of Reporting Trials
FPS-R: Faces Pain Scale-Revised
GP: general practitioner
HbA_1c_: glycated hemoglobin
ICT: information and communication technology
iTAU: improved treatment as usual
ITT: intention-to-treat
IV: instrumental variable
MBCT: mindfulness-based cognitive therapy
OFS: Openness to the Future Scale
PANAS: Positive and Negative Affect Schedule
PC: primary care
PHQ-9: Patient Health Questionnaire-9
RCT: randomized controlled trial
RM: repeated measure
RMDQ: Roland-Morris Disability Questionnaire
SF-12: 12-item Short Form Survey

## Data Availability

The datasets generated and analyzed during this study are available from the corresponding author on reasonable request.
