# Supplementary material for: Efficacy of a Blended Low-Intensity Internet-Delivered Psychological Program in Patients With Multimorbidity in Primary Care: Randomized Controlled Trial
Source: J Med Internet Res. 2025 Feb 10;27:e56203. doi: 10.2196/56203 (PMC11851034; doi:10.2196/56203)
Supplement: Multimedia Appendix 1 [file jmir_v27i1e56203_app1.docx]

**Supplement 1.** Variables at baseline as predictors of missingness

|  | Lost at postintervention | Lost at 3-month follow-up |
| --- | --- | --- |
| Socio-demographic data |  |  |
| Age | **1.04 (1.01, 1.07)^a^** | **1.04 (1.01, 1.07)** |
| Gender, *females* | 0.89 (0.46, 1.71) | 0.96 (0.50, 1.84) |
| Marital status  *Married/Relationship*  *Single*  *Separated/Divorced*  *Widowed* | ref  0.91 (0.42, 1.98)  **3.70 (1.40, 9.78)**  0.41 (0.10, 1.68) | ref  1.47 (0.67, 3.21)  **5.97 (2.25, 15.86)**  1.04 (0.28, 3.90) |
| Place of residence  *Own home*  *Relative’s home*  *Other* | ref  3.24 (0.65, 16.08)  1.16 (0.43, 3.09) | ref  2.34 (0.56, 9.69)  1.17 (0.44, 3.11) |
| Education^b^  No studies  Primary studies  Secondary studies  Tertiary studies | ref  2.25 (0.40, 12.80)  1.21 (0.23, 6.33)  0.65 (0.12, 3.63) | ref  1.79 (0.32, 10.06)  0.63 (0.12, 3.30)  1.05 (0.19, 5.78) |
| Employment^b^  Unemployed  Employed  Home duties  Sick leave  Retired | ref  0.59 (0.28, 1.24)  0.47 (0.95, 2.32)  0.55 (0.22, 1.36)  1.43 (0.50, 4.08) | ref  **0.42 (0.20, 0.90)**  **0.10 (0.01, 0.93)**  **0.27 (0.10, 0.70)**  1.17 (0.42, 3.26) |
| Income  ≤ NMW^c^  1-2 x NMW  2-4 x NMW  > 4 x NMW | ref  1.01 (0.46, 2.19)  0.87 (0.38, 1.97)  1.20 (0.18, 7.89) | ref  0.70 (0.32, 1.51)  0.88 (0.39, 1.98)  1.31 (0.20, 8.62) |
| Trial arm, *intervention + iTAU* | **1.98 (1.10, 3.57)** | 1.28 (0.71, 2.28) |
| Clinical data |  |  |
| Diagnosis, *diabetes* | **13.17 (4.90, 35.32)** | **2.24 (1.15, 4.37)** |
| Number of medications^b^ | 1.10 (0.84, 1.43) | 0.89 (0.68, 1.17) |
| Analgesics, *yes* | **0.29 (0.15, 0.57)** | **0.46 (0.25, 0.86)** |
| Antidepressants, *yes* | 0.54 (0.30, 1.00) | 0.58 (0.32, 1.07) |
| Antiepileptics, *yes* | 0.42 (0.17, 1.05) | **0.29 (0.10, 0.81)** |
| Antidiabetics, *yes* | **11.19 (4.14, 30.25)** | **2.07 (1.03, 4.15)** |
| Antithyroid, *yes* | 1.81 (0.44, 5.50) | 0.58 (0.14, 2.39) |
| Anxiolytics, *yes* | 0.72 (0.40, 1.32) | 0.71 (0.39, 1.30) |
| Cardiovascular medication, *yes* | **3.41 (1.77, 6.57)** | 1.62 (0.87, 3.00) |
| Corticosteroids, *yes* | 0.51 (0.12, 2.21) | 1.20 (0.29, 4.96) |
| Insulin, *yes* | **4.82 (1.02, 22.68)** | **3.90 (1.02, 14.94)** |
| Other, *yes* | 0.90 (0.49, 1.66) | 1.20 (0.65, 2.22) |
| Composite | 1.14 (0.82, 1.59) | 1.26 (0.89, 1.79) |
| PHQ-9^d^ | 0.99 (0.93, 1.04) | 1.02 (0.97, 1.08) |
| RMDQ^e^ | 1.07 (1.00, 1.15) | 1.02 (0.95, 1.09) |
| FPS-R^f^ | 1.10 (0.93, 1.30) | 1.07 (0.90, 1.26) |
| HbA1c^g^ | 19.53 (0.71, 535.07) | 1.23 (0.72, 2.09) |
| SF-12^h^ | 1.00 (0.98, 1.01) | 0.99 (0.97, 1.01) |
| PANAS positive^i^ | 0.99 (0.94, 1.04) | 0.99 (0.94, 1.04) |
| PANAS negative^j^ | 1.00 (0.96, 1.04) | 0.98 (0.94, 1.02) |
| OFS^k^ | 1.00 (0.96, 1.04) | 1.00 (0.96, 1.04) |

^a^In bold, effects that remained significant (P < .05).

^b^‘Neighbour/friend’s home’, ‘student’, ‘antipsychotics’, ‘NSAIDs’ were not included owing to low numbers.

^c^NMW: national minimum wage.

^d^PHQ-9: Patient Health Questionnaire.

^e^RMDQ: Rolland-Morris Scale.

^f^FPS-R: Faces Pain Scale-Revised.

^g^HbA1c: Glycosylated haemoglobin.

^h^SF-12: health-related quality of life.

^i^PANAS positive: Positive and Negative Affect Scale-positive.

^j^PANAS negative: Positive and Negative Affect Scale-negative.

^k^OFS: Openness to the Future Scale.
