# Supplementary material for: Efficacy of a Blended Low-Intensity Internet-Delivered Psychological Program in Patients With Multimorbidity in Primary Care: Randomized Controlled Trial
Source: J Med Internet Res. 2025 Feb 10;27:e56203. doi: 10.2196/56203 (PMC11851034; doi:10.2196/56203)
Supplement: Multimedia Appendix 2 [file jmir_v27i1e56203_app2.docx]

**Supplement 2.** Descriptive statistics and main comparisons at post-intervention

|  |  |  |  | Within-group | | | Between-group | | |
| --- | --- | --- | --- | --- | --- | --- | --- | --- | --- |
|  | n | Intervention + iTAU (a)  M (SD) | iTAU alone (b)  M (SD) | (a)  *B* (95% CI) | (b)  *B* (95% CI) | *d* | | *t* (*P*)^a^ | *B* (95% CI) |
| Primary outcome |  |  |  |  |  | |  |  |  |
| Composite (range: -2.25, 2.25) | 35, 49 |  |  |  |  | |  |  |  |
| Baseline |  | -0.02 (0.78) | -0.07 (0.83) |  |  | |  |  |  |
| Post-intervention |  | -0.40 (1.04) | 0.25 (0.88) | -0.39 (-0.68, -0.10) | 0.32 (0.10, 0.53) | | -0.85 | -3.93 (<.001) | -0.63 (-0.94, -0.31) |
| Secondary outcomes |  |  |  |  |  | |  |  |  |
| PHQ-9 (range: 0, 27) | 47, 65 |  |  |  |  | |  |  |  |
| Baseline |  | 14.32 (5.78) | 13.48 (5.82) |  |  | |  |  |  |
| Post-intervention |  | 8.72 (6.57) | 13.32 (6.31) | -5.40 (-7.08, -3.73) | -0.30 (-1.70, 1.10) | | -0.93 | -5.59 (<.001) | -5.02 (-6.77, -3.26) |
| RMDQ (range: 0, 24) | 33, 47 |  |  |  |  | |  |  |  |
| Baseline |  | 12.55 (5.45) | 13.66 (6.28) |  |  | |  |  |  |
| Post-intervention |  | 10.88 (6.68) | 14.30 (6.04) | -1.92 (-3.40, -0.45) | 0.48 (-0.91, 1.87) | | -0.38 | -2.46 (.014) | -2.28 (-4.10, -0.46) |
| FPS-R (range: 0, 10) | 33, 46 |  |  |  |  | |  |  |  |
| Baseline |  | 4.91 (1.81) | 4.74 (2.12) |  |  | |  |  |  |
| Post-intervention |  | 4.39 (2.74) | 5.43 (1.82) | -0.58 (-1.43, 0.27) | 0.50 (-0.07, 1.07) | | -0.60 | -1.89 (.058) | -0.96 (-1.95, 0.03) |
| HbA1c (Diabetes: ≥6.5) | 23, 14 |  |  |  |  | |  |  |  |
| Baseline |  | 6.60 (0.40) | 6.23 (0.81) |  |  | |  |  |  |
| Post-intervention |  | 6.37 (0.21) | 6.17 (0.83) | -0.24 (-0.42, -0.06) | -0.07 (-0.18, 0.05) | | -0.21 | -0.14 (.890) | -0.09 (-1.34, 1.16) |
| SF-12 (range: 0, 100) | 47, 63 |  |  |  |  | |  |  |  |
| Baseline |  | 32.79 (19.52) | 34.91 (18.03) |  |  | |  |  |  |
| Post-intervention |  | 46.90 (25.80) | 34.39 (19.63) | 15.05 (9.26, 20.84) | 0.34 (-3.08, 3.77) | | 0.78 | 4.59 (<.001) | 13.55 (7.77, 19.33) |
| PANAS positive (range: 10, 50) | 45, 63 |  |  |  |  | |  |  |  |
| Baseline |  | 20.73 (6.79) | 21.78 (7.41) |  |  | |  |  |  |
| Post-intervention |  | 24.89 (10.54) | 20.46 (7.23) | 4.33 (1.67, 6.98) | -0.97 (-2.51, 0.57) | | 0.76 | 3.83 (<.001) | 5.25 (2.56, 7.93) |
| PANAS negative (range: 10, 50) | 45, 63 |  |  |  |  | |  |  |  |
| Baseline |  | 25.98 (8.16) | 26.46 (8.56) |  |  | |  |  |  |
| Post-intervention |  | 20.13 (8.01) | 26.16 (8.78) | -6.11 (-8.40, -3.83) | -0.12 (-2.34, 2.11) | | -0.66 | -3.70 (<.001) | -5.52 (-8.45, -2.60) |
| OFS (range: 10, 50) | 47, 63 |  |  |  |  | |  |  |  |
| Baseline |  | 31.47 (7.32) | 30.73 (7.81) |  |  | |  |  |  |
| Post-intervention |  | 34.17 (8.34) | 30.16 (8.61) | 2.92 (0.83, 5.02) | -0.29 (-1.98, 1.40) | | 0.43 | 2.46 (.014) | 3.16 (0.64, 5.67) |

^a^All significant results remained significant after correction for multiple comparisons using the Benjamini-Hochberg method.

^b^PHQ-9: Patient Health Questionnaire.

^c^RMDQ: Rolland-Morris Scale.

^d^FPS-R: Faces Pain Scale-Revised.

^e^HbA1c: glycosylated haemoglobin.

^f^SF-12: Health-related quality of life.

^g^PANAS p: Positive and Negative Affect Scale-positive.

^h^PANAS n: Positive and Negative Affect Scale-negative.

^i^OFS: Openness to the Future Scale.
