# Supplementary material for: Efficacy of a Blended Low-Intensity Internet-Delivered Psychological Program in Patients With Multimorbidity in Primary Care: Randomized Controlled Trial
Source: J Med Internet Res. 2025 Feb 10;27:e56203. doi: 10.2196/56203 (PMC11851034; doi:10.2196/56203)
Supplement: Multimedia Appendix 3 [file jmir_v27i1e56203_app3.pdf]

CONSORT-EHEALTH (V 1.6.1) - Submission/Publication Form

The CONSORT-EHEALTH checklist is intended for authors of randomized trials evaluating web-based and Internet-based applications/interventions, including mobile interventions, electronic games (incl multiplayer games), social media, certain telehealth applications, and other interactive and/or networked electronic applications. Some of the items (e.g. all subitems under item 5 - description of the intervention) may also be applicable for other study designs.

The goal of the CONSORT EHEALTH checklist and guideline is to be
a) a guide for reporting for authors of RCTs,
b) to form a basis for appraisal of an ehealth trial (in terms of validity)

CONSORT-EHEALTH items/subitems are MANDATORY reporting items for studies published in the Journal of Medical Internet Research and other journals / scientific societies endorsing the checklist.

Items numbered 1., 2., 3., 4a., 4b etc are original CONSORT or CONSORT-NPT (non-pharmacologic treatment) items.
Items with Roman numerals (i., ii, iii, iv etc.) are CONSORT-EHEALTH extensions/clarifications.

As the CONSORT-EHEALTH checklist is still considered in a formative stage, we would ask that you also RATE ON A SCALE OF 1-5 how important/useful you feel each item is FOR THE PURPOSE OF THE CHECKLIST and reporting guideline (optional).

Mandatory reporting items are marked with a red *.
In the textboxes, either copy & paste the relevant sections from your manuscript into this form - please include any quotes from your manuscript in QUOTATION MARKS,
or answer directly by providing additional information not in the manuscript, or elaborating on why the item was not relevant for this study.

YOUR ANSWERS WILL BE PUBLISHED AS A SUPPLEMENTARY FILE TO YOUR PUBLICATION IN JMIR AND ARE CONSIDERED PART OF YOUR PUBLICATION (IF ACCEPTED).
Please fill in these questions diligently. Information will not be copyedited, so please use proper spelling and grammar, use correct capitalization, and avoid abbreviations.

DO NOT FORGET TO SAVE AS PDF _AND_ CLICK THE SUBMIT BUTTON SO YOUR ANSWERS ARE IN OUR DATABASE !!!

Citation Suggestion (if you append the pdf as Appendix we suggest to cite this paper in the caption):
Eysenbach G, CONSORT-EHEALTH Group
CONSORT-EHEALTH: Improving and Standardizing Evaluation Reports of Web-based and Mobile Health Interventions
J Med Internet Res 2011;13(4):e126
URL: http://www.jmir.org/2011/4/e126/
doi: 10.2196/jmir.1923
PMID: 22209829

unizar.aliciamonreal@gmail.com
Cambiar de cuenta

Borrador no guardado

No compartido

* Indica que la pregunta es obligatoria

Your name *
First Last

Alicia Monreal-Bartolomé

Primary Affiliation (short), City, Country *
University of Toronto, Toronto, Canada

University of Zaragoza, Zaragoza, Spain

Your e-mail address *
abc@gmail.com

aliciamonbart@usal.es

Title of your manuscript *

Provide the (draft) title of your manuscript.

Efficacy of a blended low-intensity internet-delivered psychological programme in patients with multimorbidity in primary care: a randomized controlled trial

Name of your App/Software/Intervention *

If there is a short and a long/alternate name, write the short name first and add the long name in brackets.

Atencion Primaria Online [Blended low-intensit

Evaluated Version (if any)

e.g. "V1", "Release 2017-03-01", "Version 2.0.27913"

Tu respuesta

Language(s) *

What language is the intervention/app in? If multiple languages are available, separate by comma (e.g. "English, French")

Spanish

URL of your Intervention Website or App

e.g. a direct link to the mobile app on app in appstore (itunes, Google Play), or URL of the website. If the intervention is a DVD or hardware, you can also link to an Amazon page.

https://atencionprimariaonline.com

URL of an image/screenshot (optional)

Tu respuesta

Accessibility *

Can an enduser access the intervention presently?

- ○ access is free and open
- ◉ access only for special usergroups, not open
- ○ access is open to everyone, but requires payment/subscription/in-app purchases
- ○ app/intervention no longer accessible
- ○ Otro:

Primary Medical Indication/Disease/Condition *

e.g. "Stress", "Diabetes", or define the target group in brackets after the condition, e.g. "Autism (Parents of children with)", "Alzheimers (Informal Caregivers of)"

Multimorbidity: depression and either type 2 di

Primary Outcomes measured in trial *

comma-separated list of primary outcomes reported in the trial

Standardized composite multimorbidity score

Secondary/other outcomes

Are there any other outcomes the intervention is expected to affect?

Severity of depressive symptoms (PHQ-9), Diabetes control (HbA1c), Pain intensity (FPS-R), Physical disability (RMDQ), Perceived health status (SF-12), Positive and Negative Affect Schedule (PANAS), Openness to the Future Scale (OFS).

Recommended "Dose" *

What do the instructions for users say on how often the app should be used?

- ○ Approximately Daily
- ◉ Approximately Weekly
- ○ Approximately Monthly
- ○ Approximately Yearly
- ○ "as needed"
- ○ Otro:

Approx. Percentage of Users (starters) still using the app as recommended after 3 months *

- (x) unknown / not evaluated
- ( ) 0-10%
- ( ) 11-20%
- ( ) 21-30%
- ( ) 31-40%
- ( ) 41-50%
- ( ) 51-60%
- ( ) 61-70%
- ( ) 71%-80%
- ( ) 81-90%
- ( ) 91-100%
- ( ) Otro:

Overall, was the app/intervention effective? *

- (selected) yes: all primary outcomes were significantly better in intervention group vs control
- partly: SOME primary outcomes were significantly better in intervention group vs control
- no statistically significant difference between control and intervention
- potentially harmful: control was significantly better than intervention in one or more outcomes
- inconclusive: more research is needed
- Otro:

Article Preparation Status/Stage *

At which stage in your article preparation are you currently (at the time you fill in this form)

- not submitted yet - in early draft status
- not submitted yet - in late draft status, just before submission
- (selected) submitted to a journal but not reviewed yet
- submitted to a journal and after receiving initial reviewer comments
- submitted to a journal and accepted, but not published yet
- published
- Otro:

Journal *

If you already know where you will submit this paper (or if it is already submitted), please provide the journal name (if it is not JMIR, provide the journal name under "other")

- ○ not submitted yet / unclear where I will submit this
- ◉ Journal of Medical Internet Research (JMIR)
- ○ JMIR mHealth and UHealth
- ○ JMIR Serious Games
- ○ JMIR Mental Health
- ○ JMIR Public Health
- ○ JMIR Formative Research
- ○ Other JMIR sister journal
- ○ Otro:

Is this a full powered effectiveness trial or a pilot/feasibility trial? *

- ○ Pilot/feasibility
- ◉ Fully powered

Manuscript tracking number *

If this is a JMIR submission, please provide the manuscript tracking number under "other" (The ms tracking number can be found in the submission acknowledgement email, or when you login as author in JMIR. If the paper is already published in JMIR, then the ms tracking number is the four-digit number at the end of the DOI, to be found at the bottom of each published article in JMIR)

- ( ) no ms number (yet) / not (yet) submitted to / published in JMIR
- (•) Otro: ms#56203

TITLE AND ABSTRACT

1a) TITLE: Identification as a randomized trial in the title

1a) Does your paper address CONSORT item 1a? *

I.e does the title contain the phrase "Randomized Controlled Trial"? (if not, explain the reason under "other")

- (•) yes
- ( ) Otro:

1a-i) Identify the mode of delivery in the title

Identify the mode of delivery. Preferably use “web-based” and/or “mobile” and/or “electronic game” in the title. Avoid ambiguous terms like “online”, “virtual”, “interactive”. Use “Internet-based” only if Intervention includes non-web-based Internet components (e.g. email), use “computer-based” or “electronic” only if offline products are used. Use “virtual” only in the context of “virtual reality” (3-D worlds). Use “online” only in the context of “online support groups”. Complement or substitute product names with broader terms for the class of products (such as “mobile” or “smart phone” instead of “iphone”), especially if the application runs on different platforms.

	1	2	3	4	5	
subitem not at all important	○	○	○	○	◉	essential

Borrar selección

Does your paper address subitem 1a-i? *

Copy and paste relevant sections from manuscript title (include quotes in quotation marks "like this" to indicate direct quotes from your manuscript), or elaborate on this item by providing additional information not in the ms, or briefly explain why the item is not applicable/relevant for your study

Yes, it is specified: "internet-delivered psychological programme"

1a-ii) Non-web-based components or important co-interventions in title

Mention non-web-based components or important co-interventions in title, if any (e.g., “with telephone support”).

	1	2	3	4	5	
subitem not at all important	○	○	○	◉	○	essential

Borrar selección

Does your paper address subitem 1a-ii?

Copy and paste relevant sections from manuscript title (include quotes in quotation marks "like this" to indicate direct quotes from your manuscript), or elaborate on this item by providing additional information not in the ms, or briefly explain why the item is not applicable/relevant for your study

Due to the length of the title, only "blended low-intensity" is specified

1a-iii) Primary condition or target group in the title

Mention primary condition or target group in the title, if any (e.g., “for children with Type I Diabetes”) Example: A Web-based and Mobile Intervention with Telephone Support for Children with Type I Diabetes: Randomized Controlled Trial

	1	2	3	4	5	
subitem not at all important	○	○	○	○	●	essential

Borrar selección

Does your paper address subitem 1a-iii? *

Copy and paste relevant sections from manuscript title (include quotes in quotation marks "like this" to indicate direct quotes from your manuscript), or elaborate on this item by providing additional information not in the ms, or briefly explain why the item is not applicable/relevant for your study

Yes, it is stated that the target group is: "patients with multimorbidity in primary care"

1b) ABSTRACT: Structured summary of trial design, methods, results, and conclusions

NPT extension: Description of experimental treatment, comparator, care providers, centers, and blinding status.

1b-i) Key features/functionalities/components of the intervention and comparator in the METHODS section of the ABSTRACT

Mention key features/functionalities/components of the intervention and comparator in the abstract. If possible, also mention theories and principles used for designing the site. Keep in mind the needs of systematic reviewers and indexers by including important synonyms. (Note: Only report in the abstract what the main paper is reporting. If this information is missing from the main body of text, consider adding it)

	1	2	3	4	5	
subitem not at all important	○	○	○	○	◉	essential

Borrar selección

Does your paper address subitem 1b-i? *

Copy and paste relevant sections from the manuscript abstract (include quotes in quotation marks "like this" to indicate direct quotes from your manuscript), or elaborate on this item by providing additional information not in the ms, or briefly explain why the item is not applicable/relevant for your study

Yes, it mention key components of the intervention and comparator: "improved treatment as usual (iTAU) combined with a blended, low-intensity psychological intervention delivered using information and communication technologies..." ... "were randomized to 'Intervention + iTAU' (combining a face-to-face intervention with a supporting online programme), or 'iTAU' alone".

1b-ii) Level of human involvement in the METHODS section of the ABSTRACT

Clarify the level of human involvement in the abstract, e.g., use phrases like “fully automated” vs. “therapist/nurse/care provider/physician-assisted” (mention number and expertise of providers involved, if any). (Note: Only report in the abstract what the main paper is reporting. If this information is missing from the main body of text, consider adding it)

	1	2	3	4	5	
subitem not at all important	○	○	○	◉	○	essential

Borrar selección

Does your paper address subitem 1b-ii?

Copy and paste relevant sections from the manuscript abstract (include quotes in quotation marks "like this" to indicate direct quotes from your manuscript), or elaborate on this item by providing additional information not in the ms, or briefly explain why the item is not applicable/relevant for your study

Yes, but not completely (lack of mention in the abstract, number and expertise of providers involved): "blended, low-intensity psychological intervention delivered using information and communication technologies", "‘Intervention + iTAU’ (combining a face-to-face intervention with a supporting online programme)".

1b-iii) Open vs. closed, web-based (self-assessment) vs. face-to-face assessments in the METHODS section of the ABSTRACT

Mention how participants were recruited (online vs. offline), e.g., from an open access website or from a clinic or a closed online user group (closed usergroup trial), and clarify if this was a purely web-based trial, or there were face-to-face components (as part of the intervention or for assessment). Clearly say if outcomes were self-assessed through questionnaires (as common in web-based trials). Note: In traditional offline trials, an open trial (open-label trial) is a type of clinical trial in which both the researchers and participants know which treatment is being administered. To avoid confusion, use “blinded” or “unblinded” to indicated the level of blinding instead of “open”, as “open” in web-based trials usually refers to “open access” (i.e. participants can self-enrol). (Note: Only report in the abstract what the main paper is reporting. If this information is missing from the main body of text, consider adding it)

	1	2	3	4	5	
subitem not at all important	○	○	○	●	○	essential

Borrar selección

Does your paper address subitem 1b-iii?

Copy and paste relevant sections from the manuscript abstract (include quotes in quotation marks "like this" to indicate direct quotes from your manuscript), or elaborate on this item by providing additional information not in the ms, or briefly explain why the item is not applicable/relevant for your study

Not fully addressed: "A two-armed, parallel group, superiority randomized controlled trial was designed for this study", "‘Intervention + iTAU’ (combining a face-to-face intervention with a supporting online programme), or ‘iTAU’ alone".

1b-iv) RESULTS section in abstract must contain use data

Report number of participants enrolled/assessed in each group, the use/uptake of the intervention (e.g., attrition/adherence metrics, use over time, number of logins etc.), in addition to primary/secondary outcomes. (Note: Only report in the abstract what the main paper is reporting. If this information is missing from the main body of text, consider adding it)

	1	2	3	4	5	
subitem not at all important	○	○	○	●	○	essential

Borrar selección

Does your paper address subitem 1b-iv?

Copy and paste relevant sections from the manuscript abstract (include quotes in quotation marks "like this" to indicate direct quotes from your manuscript), or elaborate on this item by providing additional information not in the ms, or briefly explain why the item is not applicable/relevant for your study

Yes, but not fully addressed (attrition/adherence metrics are not included in the abstract, but are briefly included in the main body of text, as they are not core to this manuscript and will be part of a different article): "Participants ... (n=183) were randomized to 'Intervention + iTAU' ..., or 'iTAU' alone", "The main outcome consisted of a standardized composite score...", "At three-month follow-up, the Intervention + iTAU group (vs iTAU) exhibited greater reductions in composite multimorbidity score (B = -0.34; 95% CI = -0.64, -0.04; g = 0.39), as well as in depression and negative affect, and improvements in perceived health, positive affect and openness to the future. Similar positive effects were observed post-intervention, also with improvements in physical disability. No significant differences were found in glycosylated haemoglobin, pain intensity or disability at three-month follow-up. Path analyses indicated a significant impact from the intervention on the primary outcome and mediated by positive and negative affect"

1b-v) CONCLUSIONS/DISCUSSION in abstract for negative trials

Conclusions/Discussions in abstract for negative trials: Discuss the primary outcome - if the trial is negative (primary outcome not changed), and the intervention was not used, discuss whether negative results are attributable to lack of uptake and discuss reasons. (Note: Only report in the abstract what the main paper is reporting. If this information is missing from the main body of text, consider adding it)

	1	2	3	4	5	
subitem not at all important	○	○	○	●	○	essential

Borrar selección

Does your paper address subitem 1b-v?

Copy and paste relevant sections from the manuscript abstract (include quotes in quotation marks "like this" to indicate direct quotes from your manuscript), or elaborate on this item by providing additional information not in the ms, or briefly explain why the item is not applicable/relevant for your study

Yes: "The present study supports the efficacy of a low-intensity psychological intervention applied in a blended format on multimorbidity in PC. It justifies exploration of the conceptualization of depression in type 2 diabetes, as well as analysis of the implementation of such interventions in routine clinical practice."

INTRODUCTION

2a) In INTRODUCTION: Scientific background and explanation of rationale

2a-i) Problem and the type of system/solution

Describe the problem and the type of system/solution that is object of the study: intended as stand-alone intervention vs. incorporated in broader health care program? Intended for a particular patient population? Goals of the intervention, e.g., being more cost-effective to other interventions, replace or complement other solutions? (Note: Details about the intervention are provided in “Methods” under 5)

	1	2	3	4	5	
subitem not at all important	○	○	○	○	◉	essential

Borrar selección

Does your paper address subitem 2a-i? *

Copy and paste relevant sections from the manuscript (include quotes in quotation marks "like this" to indicate direct quotes from your manuscript), or elaborate on this item by providing additional information not in the ms, or briefly explain why the item is not applicable/relevant for your study

Yes: "Multimorbidity (i.e., the presence of two or more chronic medical conditions) is a highly prevalent phenomenon (1–4) that has been increasing in recent decades (5–8). Its presence causes significant physical, psychological and economic impact and hinders help-seeking, diagnosis, the quality of care received and adherence to treatment (9–13).", "Comorbidity between depression and chronic medical conditions is one of the leading global public health priorities (16).", "This study focuses on two physical conditions that are comorbid with depression and that involve the greatest disability, loss in quality of life and higher health care costs: diabetes and chronic pain", "As pointed out by a previous meta-analysis, it is difficult to improve outcomes in people suffering from multimorbidity, although interventions oriented toward specific difficulties and risk factors, such as depression, are promising (16)", "The aim of this study was to evaluate the effectiveness of a blended, low-intensity psychological intervention delivered via ICTs for the treatment of multimorbidity (depression plus either type 2 diabetes or low back pain) in primary care (PC) settings. Our hypothesis was that the improved treatment-as-usual intervention, enhanced by the delivery of ICT-based, low-intensity psychological therapy, would be more effective for ameliorating multimorbidity symptoms in PC compared to a group receiving only improved treatment as usual at three months after completion of the programme".

2a-ii) Scientific background, rationale: What is known about the (type of) system

Scientific background, rationale: What is known about the (type of) system that is the object of the study (be sure to discuss the use of similar systems for other conditions/diagnoses, if appropiate), motivation for the study, i.e. what are the reasons for and what is the context for this specific study, from which stakeholder viewpoint is the study performed, potential impact of findings [2]. Briefly justify the choice of the comparator.

	1	2	3	4	5	
subitem not at all important	○	○	○	○	◉	essential

Borrar selección

Does your paper address subitem 2a-ii? *

Copy and paste relevant sections from the manuscript (include quotes in quotation marks "like this" to indicate direct quotes from your manuscript), or elaborate on this item by providing additional information not in the ms, or briefly explain why the item is not applicable/relevant for your study

Yes: "This study focuses on two physical conditions that are comorbid with depression and that involve the greatest disability, loss in quality of life and higher health care costs: diabetes and chronic pain", "As pointed out by a previous meta-analysis, it is difficult to improve outcomes in people suffering from multimorbidity, although interventions oriented toward specific difficulties and risk factors, such as depression, are promising (16)", "Patient-oriented approaches, interventions to support self-management, and training for healthcare professionals appear to be the most frequent elements of interventions with the potential to have a positive impact on patients with multimorbidity (25).", "Interventions involving the use of information and communication technologies (ICTs) have been suggested as a promising resource for the provision of adequate and timely support for the self-management of multimorbidity (26). A number of studies have shown the effectiveness of using personalized, ICT-based interventions for the treatment of depression (27), but without evaluating either their effectiveness or cost-effectiveness with regard to multimorbidity", "Other studies focusing on comorbidity deal with only one priority condition over another, instead of addressing multimorbidity (28,29). As a result, they neglect the bidirectional relationship between the different chronic conditions present and their role or influence on the course of the index disease. This approach goes against the general recommendation of simultaneously managing patients' comorbidities to forestall functionality restrictions and subsequent decline."

2b) In INTRODUCTION: Specific objectives or hypotheses

Does your paper address CONSORT subitem 2b? *

Copy and paste relevant sections from the manuscript (include quotes in quotation marks "like this" to indicate direct quotes from your manuscript), or elaborate on this item by providing additional information not in the ms, or briefly explain why the item is not applicable/relevant for your study

Yes: "The aim of this study was to evaluate the effectiveness of a blended, low-intensity psychological intervention delivered via ICTs for the treatment of multimorbidity (depression plus either type 2 diabetes or low back pain) in primary care (PC) settings. Our hypothesis was that the improved treatment-as-usual intervention, enhanced by the delivery of ICT-based, low-intensity psychological therapy, would be more effective for ameliorating multimorbidity symptoms in PC compared to a group receiving only improved treatment as usual at three months after completion of the programme".

METHODS

3a) Description of trial design (such as parallel, factorial) including allocation ratio

Does your paper address CONSORT subitem 3a? *

Copy and paste relevant sections from the manuscript (include quotes in quotation marks "like this" to indicate direct quotes from your manuscript), or elaborate on this item by providing additional information not in the ms, or briefly explain why the item is not applicable/relevant for your study

Yes: "This was a parallel group, superiority randomized controlled trial (RCT) in which patients receiving treatment as usual by their general practitioners (GPs) were randomized to receive either a) improved treatment as usual (iTAU) or b) the same iTAU combined with a blended, low-intensity, Internet-delivered psychological intervention, which comprised two individual face-to-face sessions and six individual online therapeutic modules.", "we required 180 participants in total (90 in each arm, with approximately 30 patients suffering from comorbid depression and type 2 diabetes, and 60 patients suffering from comorbid depression and low back pain)", "Randomization was performed in blocks of patients based on the PC centre and comorbid disease (i.e., type 2 diabetes or low back pain)."

3b) Important changes to methods after trial commencement (such as eligibility criteria), with reasons

Does your paper address CONSORT subitem 3b? *

Copy and paste relevant sections from the manuscript (include quotes in quotation marks "like this" to indicate direct quotes from your manuscript), or elaborate on this item by providing additional information not in the ms, or briefly explain why the item is not applicable/relevant for your study

Yes, but the third change was made before trial commencement, after the protocol was published: "Upon implementation of the protocol and commencement of the RCT, and ultimately due to the outbreak of the COVID-19 pandemic, the RCT management group was compelled to make several changes to the original study protocol (30). Three major changes were made to the original protocol (30), all of which were discussed and agreed by the trial management group prior to their implementation and were approved by the research ethics committee of the regional health authority of Aragon (CEICA; PI16/0259). Changes were made to (i) the number of participants recruited, (ii) the time point measurements, and (iii) the mechanistic outcome measures. These changes are addressed in the corresponding sections."
"The protocol (30) initially stated that the study would require 63 participants in each arm in order to detect an expected medium effect size on multimorbidity symptoms (33,34). Nevertheless, for the purpose of providing enough statistical power to compare the subjects receiving psychological therapy vs those only receiving iTAU, considering the comorbid disease that manifested in conjunction with depression (i.e., type 2 diabetes or low back pain), four possible subgroups were secondarily established, giving a total of 252 subjects. In addition, an experimental mortality of around 15% was expected (35), which meant that the required sample size was initially estimated at approximately 300 subjects. However, during the trial set-up process, difficulties emerged when it came to recruiting patients suffering from type 2 diabetes. Specifically, most type 2 diabetes patients were found to be elderly and did not have access to the Internet or e-mail, nor did they have the basic computer skills that would allow them to participate in the study. This considerably slowed down the recruitment phase of the trial. The focus on type 2 diabetes was originally decided because it is the most common type of diabetes (approximately 95%) and has a more prevalent association with major depressive disorder, and it has been more widely studied than other types of diabetes (36,37). However, given that this type of diabetes normally develops at a more advanced age and its risk increases with age, type 2 diabetes patients were much less inclined to use ICTs and thus showed greater reluctance to participate in the present study. After serious consideration, it was decided that the secondary comparison by comorbid disease subgroup would be omitted. A new sample size calculation was then performed that considered each trial arm as a whole entity.
For this purpose, we retained the possibility of detecting a similar intermediate effect size

on multimorbidity symptoms to test whether the trend in changes differed between the intervention and control groups. This criterion was considered clinically important in previous research (33,34). We also retained a power (1-ß) of .80, a significance level of 5% and a ratio of 1:1 (this time assuming a ratio between patients suffering from depression and type 2 diabetes with respect to depression and low back pain of 1:2 in each arm, owing to the previously mentioned difficulties in recruitment). According to the 'time x group' interaction in a general linear repeated measures (RMs) design with Greenhouse-Geisser correction (38), and considering a correlation across RMs that monotonically decreases with the time distance between RMs, a base correlation of 0.5 and a decay rate of 0.3, taking into account the expected 15% mortality (35), we required 180 participants in total (90 in each arm, with approximately 30 patients suffering from comorbid depression and type 2 diabetes, and 60 patients suffering from comorbid depression and low back pain)."

"During the study, a modification was made to the time point measurements. The initial protocol originally stated (30) that four timepoints would be taken (i.e., baseline, post-treatment, three-month follow-up and six-month follow-up), with the last measurement being the primary endpoint. However, the outbreak of the COVID-19 pandemic in 2020 severely affected life in Spain. As a nationwide lockdown was declared in March 2020, this resulted in PC centres being closed to the public. Spanish PC centres, health care professionals, patients and the general population were all affected by the pandemic in a range of ways – emotionally, socially and, ultimately, in terms of the health services that were provided/received. As regards the present RCT, the pandemic outbreak coincided with the final phase of the study, immediately before the last follow-up measurement. Consequently, we had completed the initial baseline measurement, as well as delivery of the intervention, post-intervention measurement and the three-month follow-up measurement, with the six-month follow-up measurement yet to be conducted. Thus, the most significant impact of the pandemic was on data collection for the primary outcome at six months. Several possibilities were considered, although they all pointed to the measurements being seriously compromised in terms of data collection and quality (e.g., uncertainty about when PC centres might reopen and social distancing issues). Consequently, the feasibility, validity and precision of the estimates – as well as the possibilities of generalizing the study results further than the specific circumstances affecting the Spanish population during the COVID-19 universal stressor pandemic – were brought into question. Therefore, it was decided that the primary endpoint would be changed from six-month follow-up to three-month follow-up after treatment, which was a measure that remained unaffected by the complex and unusual circumstances brought about by the pandemic. Ultimately, the difficult situation at the time prevented us from performing the six-month follow-up measurement."

3b-i) Bug fixes, Downtimes, Content Changes

Bug fixes, Downtimes, Content Changes: ehealth systems are often dynamic systems. A description of changes to methods therefore also includes important changes made on the intervention or comparator during the trial (e.g., major bug fixes or changes in the functionality or content) (5-iii) and other “unexpected events” that may have influenced study design such as staff changes, system failures/downtimes, etc. [2].

	1	2	3	4	5	
subitem not at all important	○	○	○	●	○	essential

Borrar selección

Does your paper address subitem 3b-i?

Copy and paste relevant sections from the manuscript (include quotes in quotation marks "like this" to indicate direct quotes from your manuscript), or elaborate on this item by providing additional information not in the ms, or briefly explain why the item is not applicable/relevant for your study

Since several pilot tests of the platform were carried out before the start of the RCT, no bug fixes, downtime or content changes of the platform were required.

4a) Eligibility criteria for participants

Does your paper address CONSORT subitem 4a? *

Copy and paste relevant sections from the manuscript (include quotes in quotation marks "like this" to indicate direct quotes from your manuscript), or elaborate on this item by providing additional information not in the ms, or briefly explain why the item is not applicable/relevant for your study

Yes: "Participants were recruited at PC health centres of the three Spanish autonomous communities participating in the study (Andalusia, Aragon and the Balearic Islands). The inclusion criteria were as follows: (i) minimum age of 18 years; (ii) DSM-5 diagnosis of major depression or persistent depressive disorder, of mild or moderate severity, expressed as a Patient Health Questionnaire (PHQ-9) score lower than 14; (iii) duration of depressive symptoms of two months or longer; (iv) diagnosis of one of the following two conditions: a) type 2 diabetes (diagnosis according to criteria of the American Diabetes Association [ADA] (31)) or b) low back pain (diagnosis of non-specific chronic low back pain according to the definition established by the European Cooperation in Science and Technology (COST) B-13 clinical practice guidelines (32) with a duration of at least six months); (v) possession of and ability to use a computer, an Internet connection and a mobile phone; (vi) ability to understand oral and written Spanish; and (vii) willingness to participate in the study and signing the corresponding informed consent. The following exclusion criteria were applied: (i) any diagnosis of a disease that might affect the central nervous system (e.g. brain condition, traumatic brain injury, dementia); (ii) other psychiatric diagnoses or acute mental illness (e.g. substance dependence or abuse, history of schizophrenia or other psychotic disorders, eating disorders), except for anxiety disorder or personality disorders; (iii) any medical, infectious or degenerative disease that might affect mood; (iv) presence of delusional ideas or hallucinations, whether or not consistent with mood; and (v) suicide risk."

4a-i) Computer / Internet literacy

Computer / Internet literacy is often an implicit "de facto" eligibility criterion - this should be explicitly clarified.

	1	2	3	4	5	
subitem not at all important	○	○	○	◉	○	essential

Borrar selección

Does your paper address subitem 4a-i?

Copy and paste relevant sections from the manuscript (include quotes in quotation marks "like this" to indicate direct quotes from your manuscript), or elaborate on this item by providing additional information not in the ms, or briefly explain why the item is not applicable/relevant for your study

Yes: "The inclusion criteria were as follows: ...(v) possession of and ability to use a computer, an Internet connection and a mobile phone;"

4a-ii) Open vs. closed, web-based vs. face-to-face assessments:

Open vs. closed, web-based vs. face-to-face assessments: Mention how participants were recruited (online vs. offline), e.g., from an open access website or from a clinic, and clarify if this was a purely web-based trial, or there were face-to-face components (as part of the intervention or for assessment), i.e., to what degree got the study team to know the participant. In online-only trials, clarify if participants were quasi-anonymous and whether having multiple identities was possible or whether technical or logistical measures (e.g., cookies, email confirmation, phone calls) were used to detect/prevent these.

	1	2	3	4	5	
subitem not at all important	○	○	○	○	◉	essential

Borrar selección

Does your paper address subitem 4a-ii? *

Copy and paste relevant sections from the manuscript (include quotes in quotation marks "like this" to indicate direct quotes from your manuscript), or elaborate on this item by providing additional information not in the ms, or briefly explain why the item is not applicable/relevant for your study

Yes: "Participants were recruited at PC health centres of the three Spanish autonomous communities participating in the study (Andalusia, Aragon and the Balearic Islands)."
"Patients were recruited by GPs working in PC centres of the previously mentioned Spanish autonomous communities, who subsequently sent the referral and consent forms of potential participants to the evaluating investigator. The evaluating investigator then contacted the participants to schedule the screening assessments and recorded the psychological and biological variables to determine their inclusion. Randomization was performed in blocks of patients based on the PC centre and comorbid disease (i.e., type 2 diabetes or low back pain). An independent researcher unrelated to the research team generated the individual randomization list using a randomization software. A researcher from the research team not involved in any other project-related task, together with an independent general practitioner, performed data monitoring tasks. For further information regarding masking and procedures, see the protocol (30). The participants consented to their inclusion before finding out the treatment to which they were assigned and were permitted to withdraw from treatment at any time."

4a-iii) Information giving during recruitment

Information given during recruitment. Specify how participants were briefed for recruitment and in the informed consent procedures (e.g., publish the informed consent documentation as appendix, see also item X26), as this information may have an effect on user self-selection, user expectation and may also bias results.

	1	2	3	4	5	
subitem not at all important	○	○	○	◉	○	essential

Borrar selección

Does your paper address subitem 4a-iii?

Copy and paste relevant sections from the manuscript (include quotes in quotation marks "like this" to indicate direct quotes from your manuscript), or elaborate on this item by providing additional information not in the ms, or briefly explain why the item is not applicable/relevant for your study

Yes: "Patients were recruited by GPs working in PC centres of the previously mentioned Spanish autonomous communities, who subsequently sent the referral and consent forms of potential participants to the evaluating investigator. The evaluating investigator then contacted the participants to schedule the screening assessments and recorded the psychological and biological variables to determine their inclusion. Randomization was performed in blocks of patients based on the PC centre and comorbid disease (i.e., type 2 diabetes or low back pain). An independent researcher unrelated to the research team generated the individual randomization list using a randomization software. A researcher from the research team not involved in any other project-related task, together with an independent general practitioner, performed data monitoring tasks. For further information regarding masking and procedures, see the protocol (30). The participants consented to their inclusion before finding out the treatment to which they were assigned and were permitted to withdraw from treatment at any time."
"Written informed consent was obtained before screening and the application of exclusion criteria took place. Since the study involved the use of the Internet, an important ethical issue was data protection. In order to ensure the protection of personal information, Advanced Encryption Standard (AES) strategies regarding data encryption and use of personal passwords were implemented. The data were treated anonymously and confidentially and only used for the purposes of the study."
"This study was approved by the research ethics committee in each of the autonomous communities involved (CEICA Aragon, CEIC Balearic Islands and the Regional Ethics and Research Committee of the province of Malaga) and was designed in accordance with the ethical standards laid down in the Declaration of Helsinki and its later amendments."

4b) Settings and locations where the data were collected

Does your paper address CONSORT subitem 4b? *

Copy and paste relevant sections from the manuscript (include quotes in quotation marks "like this" to indicate direct quotes from your manuscript), or elaborate on this item by providing additional information not in the ms, or briefly explain why the item is not applicable/relevant for your study

Yes, but it is explained in depth in the published protocol: "The evaluating investigator then contacted the participants to schedule the screening assessments and recorded the psychological and biological variables to determine their inclusion.", "A researcher from the research team not involved in any other project-related task, together with an independent general practitioner, performed data monitoring tasks. For further information regarding masking and procedures, see the protocol (30)."
"Since the study involved the use of the Internet, an important ethical issue was data protection. In order to ensure the protection of personal information, Advanced Encryption Standard (AES) strategies regarding data encryption and use of personal passwords were implemented. The data were treated anonymously and confidentially and only used for the purposes of the study."

4b-i) Report if outcomes were (self-)assessed through online questionnaires

Clearly report if outcomes were (self-)assessed through online questionnaires (as common in web-based trials) or otherwise.

	1	2	3	4	5	
subitem not at all important	○	○	○	○	◉	essential

Borrar selección

Does your paper address subitem 4b-i? *

Copy and paste relevant sections from the manuscript (include quotes in quotation marks "like this" to indicate direct quotes from your manuscript), or elaborate on this item by providing additional information not in the ms, or briefly explain why the item is not applicable/relevant for your study

Yes, but it is explained in depth in the published protocol: "The evaluating investigator then contacted the participants to schedule the screening assessments and recorded the psychological and biological variables to determine their inclusion.", "A researcher from the research team not involved in any other project-related task, together with an independent general practitioner, performed data monitoring tasks. For further information regarding masking and procedures, see the protocol (30)."

4b-ii) Report how institutional affiliations are displayed

Report how institutional affiliations are displayed to potential participants [on ehealth media], as affiliations with prestigious hospitals or universities may affect volunteer rates, use, and reactions with regards to an intervention.(Not a required item – describe only if this may bias results)

	1	2	3	4	5	
subitem not at all important	○	○	◉	○	○	essential

Borrar selección

Does your paper address subitem 4b-ii?

Copy and paste relevant sections from the manuscript (include quotes in quotation marks "like this" to indicate direct quotes from your manuscript), or elaborate on this item by providing additional information not in the ms, or briefly explain why the item is not applicable/relevant for your study

It is not specified in the text, but the logos of the main institutions involved in the project (funders and developers) are only displayed on the homepage at the bottom of the online platform. These institutions logos are: "Carlos III Health Institute", "The Preventive Activities and Health Promotion Research Network" and "Labpsitec".

5) The interventions for each group with sufficient details to allow replication, including how and when they were actually administered

5-i) Mention names, credential, affiliations of the developers, sponsors, and owners

Mention names, credential, affiliations of the developers, sponsors, and owners [6] (if authors/evaluators are owners or developer of the software, this needs to be declared in a “Conflict of interest” section or mentioned elsewhere in the manuscript).

	1	2	3	4	5	
subitem not at all important	○	○	○	●	○	essential

Borrar selección

Does your paper address subitem 5-i?

Copy and paste relevant sections from the manuscript (include quotes in quotation marks "like this" to indicate direct quotes from your manuscript), or elaborate on this item by providing additional information not in the ms, or briefly explain why the item is not applicable/relevant for your study

Names and affiliations of the developers (authors), as well as sponsors, are mentioned in their respective sections: "This research was financed by a FIS grant from the Carlos III Health Institute of the Spanish Ministry of Economy and Competitiveness (PI16/00962). The first author had a FI predoctoral contract awarded by the Carlos III Health Institute (ISCIII; FI17/00180) to the Institute of Health Research of Aragon (IIS), Zaragoza, in order to conduct this study. The funding body had no role in the design of the study and data collection, nor did it play any part in the analysis, interpretation of data or the writing of manuscripts. The funding entity audited the trial execution."

"Alicia Monreal-Bartolomé1,2,3, Adoración Castro4,5,6, M. Ángeles Pérez-Ara4, Margalida Gili1,4-6, Fermín Mayoral7,8, María Magdalena Hurtado7,8, Esperanza Varela Moreno1,7-9, Cristina Botella10,11, Azucena García-Palacios10,11, Rosa M. Baños10,12, Yolanda López-Del-Hoyo1,2,3, Javier García-Campayo1,2*, Jesús Montero-Marin13,14,15

1Research Network on Chronicity, Primary Care and Health Promotion RD21/0016/0005, RICAPPS, Zaragoza Spain. 2Aragon Institute for Health Research, IIS Aragon, Zaragoza, Spain. 3Department of Psychology and Sociology, University of Zaragoza, Zaragoza, Spain. 4Research Institute of Health Sciences (IUNICS), University of the Balearic Islands (UIB), Palma de Mallorca, Spain. 5Health Research Institute of the Balearic Islands (IdISBa), Hospital Universitario Son Espases, Edificio S, Palma de Mallorca, Spain. 6Department of Psychology, University of the Balearic Islands (UIB), Palma de Mallorca, Spain. 7Mental Health Department, Institute of Biomedicine of Malaga, University Regional Hospital of Malaga, Malaga, Spain. 8Biomedical Research Institute of Málaga, IBIMA, Málaga, Spain. 9Research and Innovation Unit, Costa del Sol University Hospital, Marbella, Málaga, Spain. 10CIBER Physiopathology Obesity and Nutrition (CIBERobn) Carlos III Health Institute, Madrid, Spain. 11Department of Clinical and Basic Psychology and Biopsychology, Faculty of Health Sciences, University Jaume I, Castellon, Spain. 12Department of Psychological, Personality, Evaluation and Treatment, University of Valencia, Valencia, Spain. 13Department of Psychiatry, Warneford Hospital, University of Oxford, Oxford, UK. 14Teaching, Research & Innovation Unit, Parc Sanitari Sant Joan de Déu, Sant Boi de Llobregat, Spain. 15Consortium for Biomedical Research in Epidemiology & Public Health (CIBER Epidemiology and Public Health - CIBERESP), Madrid, Spain."

5-ii) Describe the history/development process

Describe the history/development process of the application and previous formative evaluations (e.g., focus groups, usability testing), as these will have an impact on adoption/use rates and help with interpreting results.

	1	2	3	4	5	
subitem not at all important	○	○	○	◉	○	essential

Borrar selección

Does your paper address subitem 5-ii?

Copy and paste relevant sections from the manuscript (include quotes in quotation marks "like this" to indicate direct quotes from your manuscript), or elaborate on this item by providing additional information not in the ms, or briefly explain why the item is not applicable/relevant for your study

Only the components of the intervention and certain aspects of the process and functioning of the intervention are described here, as an independent article is being prepared for the adherence and usability results that will take these aspects into account. In addition, qualitative data (from individual interviews and focus groups) on usability, expectations, etc. of the platform are being analysed, the methodology and results of which will be presented in depth in a later paper.
"These modules were supported by multimedia materials (e.g., videos and audios) and had an approximate duration of 60 minutes each."... "The structure of the modules consistently followed the same pattern (30) and concluded with suggested assignments to enable the material covered to be practised. Prior to the commencement of each module, the participants were prompted to confirm their completion of the recommended assignments, and they received a response either congratulating them for or encouraging them to finish the tasks."... "To improve engagement, after a period of inactivity on the software (which was scheduled according to the patient's preferences), users received an e-mail reminder to continue completing the modules".

5-iii) Revisions and updating

Revisions and updating. Clearly mention the date and/or version number of the application/intervention (and comparator, if applicable) evaluated, or describe whether the intervention underwent major changes during the evaluation process, or whether the development and/or content was “frozen” during the trial. Describe dynamic components such as news feeds or changing content which may have an impact on the replicability of the intervention (for unexpected events see item 3b).

	1	2	3	4	5	
subitem not at all important	○	○	○	◉	○	essential

Borrar selección

Does your paper address subitem 5-iii?

Copy and paste relevant sections from the manuscript (include quotes in quotation marks "like this" to indicate direct quotes from your manuscript), or elaborate on this item by providing additional information not in the ms, or briefly explain why the item is not applicable/relevant for your study

No changes were made to the platform once it was designed and the study began (i.e. the development and content was “frozen” during the trial).

5-iv) Quality assurance methods

Provide information on quality assurance methods to ensure accuracy and quality of information provided [1], if applicable.

	1	2	3	4	5	
subitem not at all important	○	○	○	◉	○	essential

Borrar selección

Does your paper address subitem 5-iv?

Copy and paste relevant sections from the manuscript (include quotes in quotation marks "like this" to indicate direct quotes from your manuscript), or elaborate on this item by providing additional information not in the ms, or briefly explain why the item is not applicable/relevant for your study

Yes: "Since the study involved the use of the Internet, an important ethical issue was data protection. In order to ensure the protection of personal information, Advanced Encryption Standard (AES) strategies regarding data encryption and use of personal passwords were implemented. The data were treated anonymously and confidentially and only used for the purposes of the study."

5-v) Ensure replicability by publishing the source code, and/or providing screenshots/screen-capture video, and/or providing flowcharts of the algorithms used

Ensure replicability by publishing the source code, and/or providing screenshots/screen-capture video, and/or providing flowcharts of the algorithms used. Replicability (i.e., other researchers should in principle be able to replicate the study) is a hallmark of scientific reporting.

	1	2	3	4	5	
subitem not at all important	○	○	○	●	○	essential

Borrar selección

Does your paper address subitem 5-v?

Copy and paste relevant sections from the manuscript (include quotes in quotation marks "like this" to indicate direct quotes from your manuscript), or elaborate on this item by providing additional information not in the ms, or briefly explain why the item is not applicable/relevant for your study

Neither the source code nor the algorithms used are shown, since it is not the objective of this article/trial to expose these data, and furthermore, they cannot be publicly disclosed since it is a platform, for the moment private, recently created and tested in this RCT, whose open dissemination will occur once it has sufficient empirical evidence, after analyzing the data from this and other studies of viability, usability, etc. associated with it.

5-vi) Digital preservation

Digital preservation: Provide the URL of the application, but as the intervention is likely to change or disappear over the course of the years; also make sure the intervention is archived (Internet Archive, webcitation.org, and/or publishing the source code or screenshots/videos alongside the article). As pages behind login screens cannot be archived, consider creating demo pages which are accessible without login.

	1	2	3	4	5	
subitem not at all important	○	○	○	◉	○	essential

Borrar selección

Does your paper address subitem 5-vi?

Copy and paste relevant sections from the manuscript (include quotes in quotation marks "like this" to indicate direct quotes from your manuscript), or elaborate on this item by providing additional information not in the ms, or briefly explain why the item is not applicable/relevant for your study

https://atencionprimariaonline.com/

5-vii) Access

Access: Describe how participants accessed the application, in what setting/context, if they had to pay (or were paid) or not, whether they had to be a member of specific group. If known, describe how participants obtained “access to the platform and Internet” [1]. To ensure access for editors/reviewers/readers, consider to provide a “backdoor” login account or demo mode for reviewers/readers to explore the application (also important for archiving purposes, see vi).

	1	2	3	4	5	
subitem not at all important	○	○	○	○	◉	essential

Borrar selección

Does your paper address subitem 5-vii? *

Copy and paste relevant sections from the manuscript (include quotes in quotation marks "like this" to indicate direct quotes from your manuscript), or elaborate on this item by providing additional information not in the ms, or briefly explain why the item is not applicable/relevant for your study

As explained in the method, participants obtained access through the general practitioners of their health centers who offered them to participate in the study, and, if they met the selection criteria, they could participate voluntarily in the study, without being paid for it. "The inclusion criteria were as follows: (i) minimum age of 18 years; (ii) DSM-5 diagnosis of major depression or persistent depressive disorder, of mild or moderate severity, expressed as a Patient Health Questionnaire (PHQ-9) score lower than 14; (iii) duration of depressive symptoms of two months or longer; (iv) diagnosis of one of the following two conditions: a) type 2 diabetes (diagnosis according to criteria of the American Diabetes Association [ADA] (31)) or b) low back pain (diagnosis of non-specific chronic low back pain according to the definition established by the European Cooperation in Science and Technology (COST) B-13 clinical practice guidelines (32) with a duration of at least six months); (v) possession of and ability to use a computer, an Internet connection and a mobile phone; (vi) ability to understand oral and written Spanish; and (vii) willingness to participate in the study and signing the corresponding informed consent."
"Patients were recruited by GPs working in PC centres of the previously mentioned Spanish autonomous communities, who subsequently sent the referral and consent forms of potential participants to the evaluating investigator. The evaluating investigator then contacted the participants to schedule the screening assessments and recorded the psychological and biological variables to determine their inclusion" ...."For further information regarding masking and procedures, see the protocol (30). The participants consented to their inclusion before finding out the treatment to which they were assigned and were permitted to withdraw from treatment at any time."

5-viii) Mode of delivery, features/functionalities/components of the intervention and comparator, and the theoretical framework

Describe mode of delivery, features/functionalities/components of the intervention and comparator, and the theoretical framework [6] used to design them (instructional strategy [1], behaviour change techniques, persuasive features, etc., see e.g., [7, 8] for terminology). This includes an in-depth description of the content (including where it is coming from and who developed it) [1],” whether [and how] it is tailored to individual circumstances and allows users to track their progress and receive feedback” [6]. This also includes a description of communication delivery channels and – if computer-mediated communication is a component – whether communication was synchronous or asynchronous [6]. It also includes information on presentation strategies [1], including page design principles, average amount of text on pages, presence of hyperlinks to other resources, etc. [1].

	1	2	3	4	5	
subitem not at all important	○	○	○	○	◉	essential

Borrar selección

Does your paper address subitem 5-viii? *

Copy and paste relevant sections from the manuscript (include quotes in quotation marks "like this" to indicate direct quotes from your manuscript), or elaborate on this item by providing additional information not in the ms, or briefly explain why the item is not applicable/relevant for your study

Yes: "...It consisted of two face-to-face individual sessions and six online individual and interactive therapeutic modules. The online therapeutic modules were oriented to work on different psychological techniques and therapeutic strategies that have demonstrated their efficacy for treating depression, diabetes and chronic low back pain – including motivational techniques, psychoeducation on depression and healthy lifestyle, behavioural activation, positive psychology and mindfulness-based components (40–49). These modules were supported by multimedia materials (e.g., videos and audios) and had an approximate duration of 60 minutes each.
The content of the modules is summarized in Table 1. The structure of the modules consistently followed the same pattern (30) and concluded with suggested assignments to enable the material covered to be practised. Prior to the commencement of each module, the participants were prompted to confirm their completion of the recommended assignments, and they received a response either congratulating them for or encouraging them to finish the tasks. Completing these assignments is considered crucial for consolidating the knowledge acquired in the programme, and for translating the strategies of the programme into skills. To improve engagement, after a period of inactivity on the software (which was scheduled according to the patient's preferences), users received an e-mail reminder to continue completing the modules. The programme was designed with a duration of 8–12 weeks."
"All the patients included in the study (both those in the control group and those included in the intervention group) were given their usual treatment by their GPs in PC. This treatment is described as improved because the participating GPs received a training programme based on the widely used Spanish Guidelines for the Treatment of Depression in PC, which are based on the NICE guidelines (39)."
"This is the first study to employ blended models involving ICTs in the treatment of multimorbidity. It focused on a particularly prevalent, disabling and challenging condition in clinical practice – multimorbidity between depression and type 2 diabetes or chronic low back pain. Furthermore, an evidence-based design and intervention were proposed, adhering to the recommendations of major clinical guidelines and previous research. The intervention specifically targeted risk factors, such as depression, and addressed functional difficulties. It was centred on patients and their specific needs, offering support for self-management, adopting a comprehensive and personalized approach, and incorporating therapeutic objectives negotiated and reassessed throughout the process in accordance with Ariadne's principles (21)."

5-ix) Describe use parameters

Describe use parameters (e.g., intended “doses” and optimal timing for use). Clarify what instructions or recommendations were given to the user, e.g., regarding timing, frequency, heaviness of use, if any, or was the intervention used ad libitum.

	1	2	3	4	5	
subitem not at all important	○	○	○	◉	○	essential

Borrar selección

Does your paper address subitem 5-ix?

Copy and paste relevant sections from the manuscript (include quotes in quotation marks "like this" to indicate direct quotes from your manuscript), or elaborate on this item by providing additional information not in the ms, or briefly explain why the item is not applicable/relevant for your study

Yes, but more specific information is provided on the protocol paper (e.g. optimal timing for use), which is mentioned in the text: "For further information regarding masking and procedures, see the protocol (30)."
"These modules were supported by multimedia materials (e.g., videos and audios) and had an approximate duration of 60 minutes each.", "The structure of the modules consistently followed the same pattern (30) and concluded with suggested assignments to enable the material covered to be practised. Prior to the commencement of each module, the participants were prompted to confirm their completion of the recommended assignments, and they received a response either congratulating them for or encouraging them to finish the tasks. Completing these assignments is considered crucial for consolidating the knowledge acquired in the programme, and for translating the strategies of the programme into skills. To improve engagement, after a period of inactivity on the software (which was scheduled according to the patient’s preferences), users received an e-mail reminder to continue completing the modules. The programme was designed with a duration of 8–12 weeks."

5-x) Clarify the level of human involvement

Clarify the level of human involvement (care providers or health professionals, also technical assistance) in the e-intervention or as co-intervention (detail number and expertise of professionals involved, if any, as well as “type of assistance offered, the timing and frequency of the support, how it is initiated, and the medium by which the assistance is delivered”. It may be necessary to distinguish between the level of human involvement required for the trial, and the level of human involvement required for a routine application outside of a RCT setting (discuss under item 21 – generalizability).

	1	2	3	4	5	
subitem not at all important	○	○	○	●	○	essential

Borrar selección

Does your paper address subitem 5-x?

Copy and paste relevant sections from the manuscript (include quotes in quotation marks "like this" to indicate direct quotes from your manuscript), or elaborate on this item by providing additional information not in the ms, or briefly explain why the item is not applicable/relevant for your study

These aspects were described in greater depth in the study protocol, but are also addressed here: "It consisted of two face-to-face individual sessions and six online individual and interactive therapeutic modules."..."Prior to the commencement of each module, the participants were prompted to confirm their completion of the recommended assignments, and they received an automatic response either congratulating them for or encouraging them to finish the tasks." ..."To improve engagement, after a period of inactivity on the software (which was scheduled according to the patient’s preferences), users received an e-mail reminder to continue completing the modules."
Also see: Table 1. Overview of the program"

5-xi) Report any prompts/reminders used

Report any prompts/reminders used: Clarify if there were prompts (letters, emails, phone calls, SMS) to use the application, what triggered them, frequency etc. It may be necessary to distinguish between the level of prompts/reminders required for the trial, and the level of prompts/reminders for a routine application outside of a RCT setting (discuss under item 21 – generalizability).

	1	2	3	4	5	
subitem not at all important	○	○	○	○	◉	essential

Borrar selección

Does your paper address subitem 5-xi? *

Copy and paste relevant sections from the manuscript (include quotes in quotation marks "like this" to indicate direct quotes from your manuscript), or elaborate on this item by providing additional information not in the ms, or briefly explain why the item is not applicable/relevant for your study

Yes: "...they received an automatic response either congratulating them for or encouraging them to finish the tasks." ... "users received an e-mail reminder to continue completing the modules."

5-xii) Describe any co-interventions (incl. training/support)

Describe any co-interventions (incl. training/support): Clearly state any interventions that are provided in addition to the targeted eHealth intervention, as ehealth intervention may not be designed as stand-alone intervention. This includes training sessions and support [1]. It may be necessary to distinguish between the level of training required for the trial, and the level of training for a routine application outside of a RCT setting (discuss under item 21 – generalizability.

	1	2	3	4	5	
subitem not at all important	○	○	○	○	◉	essential

Borrar selección

Does your paper address subitem 5-xii? *

Copy and paste relevant sections from the manuscript (include quotes in quotation marks "like this" to indicate direct quotes from your manuscript), or elaborate on this item by providing additional information not in the ms, or briefly explain why the item is not applicable/relevant for your study

Yes: "It consisted of two face-to-face individual sessions and six online individual and interactive therapeutic modules." "The content of the modules is summarized in Table 1."

6a) Completely defined pre-specified primary and secondary outcome measures, including how and when they were assessed

Does your paper address CONSORT subitem 6a? *

Copy and paste relevant sections from the manuscript (include quotes in quotation marks "like this" to indicate direct quotes from your manuscript), or elaborate on this item by providing additional information not in the ms, or briefly explain why the item is not applicable/relevant for your study

Primary and secondary outcome measures are fully defined, but how and when they were administered was specified in the published study protocol (DOI:10.1186/s12888-019-2037-3), which is referred to in the method section for more specific information.

6a-i) Online questionnaires: describe if they were validated for online use and apply CHERRIES items to describe how the questionnaires were designed/deployed

If outcomes were obtained through online questionnaires, describe if they were validated for online use and apply CHERRIES items to describe how the questionnaires were designed/deployed [9].

	1	2	3	4	5	
subitem not at all important	○	○	○	◉	○	essential

Borrar selección

Does your paper address subitem 6a-i?

Copy and paste relevant sections from manuscript text

It was specified in the published study protocol (DOI:10.1186/s12888-019-2037-3), which is referred to in the method section for more specific information.

6a-ii) Describe whether and how “use” (including intensity of use/dosage) was defined/measured/monitored

Describe whether and how “use” (including intensity of use/dosage) was defined/measured/monitored (logins, logfile analysis, etc.). Use/adoption metrics are important process outcomes that should be reported in any ehealth trial.

	1	2	3	4	5	
subitem not at all important	○	○	○	●	○	essential

Borrar selección

Does your paper address subitem 6a-ii?

Copy and paste relevant sections from manuscript text

An independent article is being prepared for the adherence and usability results that will take these aspects into account.

6a-iii) Describe whether, how, and when qualitative feedback from participants was obtained

Describe whether, how, and when qualitative feedback from participants was obtained (e.g., through emails, feedback forms, interviews, focus groups).

	1	2	3	4	5	
subitem not at all important	○	○	○	●	○	essential

Borrar selección

Does your paper address subitem 6a-iii?

Copy and paste relevant sections from manuscript text

Qualitative data (from individual interviews and focus groups) on usability, expectations, etc. of the platform are being analysed, the methodology and results of which will be reported in depth in a later paper.

6b) Any changes to trial outcomes after the trial commenced, with reasons

Does your paper address CONSORT subitem 6b? *

Copy and paste relevant sections from the manuscript (include quotes in quotation marks "like this" to indicate direct quotes from your manuscript), or elaborate on this item by providing additional information not in the ms, or briefly explain why the item is not applicable/relevant for your study

Yes, changes were made to the original protocol all of which were discussed and agreed by the trial management group prior to their implementation and were approved by the research ethics committee of the regional health authority of Aragon (CEICA; PI16/0259): "During the study, a modification was made to the time point measurements. The initial protocol originally stated (30) that four timepoints would be taken (i.e., baseline, post treatment, three-month follow-up and six-month follow-up), with the last measurement being the primary endpoint. However, the outbreak of the COVID-19 pandemic in 2020 severely affected life in Spain. As a nationwide lockdown was declared in March 2020, this resulted in PC centres being closed to the public. Spanish PC centres, health care professionals, patients and the general population were all affected by the pandemic in a range of ways – emotionally, socially and, ultimately, in terms of the health services that were provided/received. As regards the present RCT, the pandemic outbreak coincided with the final phase of the study, immediately before the last follow-up measurement. Consequently, we had completed the initial baseline measurement, as well as delivery of the intervention, post-intervention measurement and the three-month follow-up measurement, with the six-month follow-up measurement yet to be conducted. Thus, the most significant impact of the pandemic was on data collection for the primary outcome at six months. Several possibilities were considered, although they all pointed to the measurements being seriously compromised in terms of data collection and quality (e.g., uncertainty about when PC centres might reopen and social distancing issues). Consequently, the feasibility, validity and precision of the estimates – as well as the possibilities of generalizing the study results further than the specific circumstances affecting the Spanish population during the COVID-19 universal stressor pandemic – were brought into question. Therefore, it was decided that the primary endpoint would be changed from six-month follow-up to three-month follow-up after treatment, which was a measure that remained unaffected by the complex and unusual circumstances brought about by the pandemic. Ultimately, the difficult situation at the time prevented us from performing the six-month follow-up measurement."

7a) How sample size was determined

NPT: When applicable, details of whether and how the clustering by care provides or centers was addressed

7a-i) Describe whether and how expected attrition was taken into account when calculating the sample size

Describe whether and how expected attrition was taken into account when calculating the sample size.

	1	2	3	4	5	
subitem not at all important	○	○	○	◉	○	essential

Borrar selección

Does your paper address subitem 7a-i?

Copy and paste relevant sections from manuscript title (include quotes in quotation marks "like this" to indicate direct quotes from your manuscript), or elaborate on this item by providing additional information not in the ms, or briefly explain why the item is not applicable/relevant for your study

Yes: "According to the 'time x group' interaction in a general linear repeated measures (RMs) design with Greenhouse-Geisser correction ... a base correlation of 0.5 and a decay rate of 0.3, taking into account the expected 15% mortality (35), we required 180 participants in total (90 in each arm, with approximately 30 patients suffering from comorbid depression and type 2 diabetes, and 60 patients suffering from comorbid depression and low back pain)."

7b) When applicable, explanation of any interim analyses and stopping guidelines

Does your paper address CONSORT subitem 7b? *

Copy and paste relevant sections from the manuscript (include quotes in quotation marks "like this" to indicate direct quotes from your manuscript), or elaborate on this item by providing additional information not in the ms, or briefly explain why the item is not applicable/relevant for your study

Yes: "For this purpose, we retained the possibility of detecting a similar intermediate effect size on multimorbidity symptoms to test whether the trend in changes differed between the intervention and control groups. This criterion was considered clinically important in previous research (33,34). We also retained a power (1-ß) of .80, a significance level of 5% and a ratio of 1:1 (this time assuming a ratio between patients suffering from depression and type 2 diabetes with respect to depression and low back pain of 1:2 in each arm, owing to the previously mentioned difficulties in recruitment)."
No stopping guidelines were established.

8a) Method used to generate the random allocation sequence
NPT: When applicable, how care providers were allocated to each trial group

Does your paper address CONSORT subitem 8a? *

Copy and paste relevant sections from the manuscript (include quotes in quotation marks "like this" to indicate direct quotes from your manuscript), or elaborate on this item by providing additional information not in the ms, or briefly explain why the item is not applicable/relevant for your study

Yes: "An independent researcher unrelated to the research team generated the individual randomization list using a randomization software."

8b) Type of randomisation; details of any restriction (such as blocking and block size)

Does your paper address CONSORT subitem 8b? *

Copy and paste relevant sections from the manuscript (include quotes in quotation marks "like this" to indicate direct quotes from your manuscript), or elaborate on this item by providing additional information not in the ms, or briefly explain why the item is not applicable/relevant for your study

Yes: "Randomization was performed in blocks of patients based on the PC centre and comorbid disease (i.e., type 2 diabetes or low back pain)." "For further information regarding masking and procedures, see the protocol (30)."

9) Mechanism used to implement the random allocation sequence (such as sequentially numbered containers), describing any steps taken to conceal the sequence until interventions were assigned

Does your paper address CONSORT subitem 9? *

Copy and paste relevant sections from the manuscript (include quotes in quotation marks "like this" to indicate direct quotes from your manuscript), or elaborate on this item by providing additional information not in the ms, or briefly explain why the item is not applicable/relevant for your study

Yes: "Randomization was performed in blocks of patients based on the PC centre and comorbid disease (i.e., type 2 diabetes or low back pain)."

10) Who generated the random allocation sequence, who enrolled participants, and who assigned participants to interventions

Does your paper address CONSORT subitem 10? *

Copy and paste relevant sections from the manuscript (include quotes in quotation marks "like this" to indicate direct quotes from your manuscript), or elaborate on this item by providing additional information not in the ms, or briefly explain why the item is not applicable/relevant for your study

Yes: "Patients were recruited by GPs working in PC centres of the previously mentioned Spanish autonomous communities, who subsequently sent the referral and consent forms of potential participants to the evaluating investigator. The evaluating investigator then contacted the participants to schedule the screening assessments and recorded the psychological and biological variables to determine their inclusion. Randomization was performed in blocks of patients based on the PC centre and comorbid disease (i.e., type 2 diabetes or low back pain). An independent researcher unrelated to the research team generated the individual randomization list using a randomization software. A researcher from the research team not involved in any other project-related task, together with an independent general practitioner, performed data monitoring tasks. For further information regarding masking and procedures, see the protocol (30)."

11a) If done, who was blinded after assignment to interventions (for example, participants, care providers, those assessing outcomes) and how

NPT: Whether or not administering co-interventions were blinded to group assignment

11a-i) Specify who was blinded, and who wasn't

Specify who was blinded, and who wasn't. Usually, in web-based trials it is not possible to blind the participants [1, 3] (this should be clearly acknowledged), but it may be possible to blind outcome assessors, those doing data analysis or those administering co-interventions (if any).

	1	2	3	4	5	
subitem not at all important	○	○	○	○	◉	essential

Borrar selección

Does your paper address subitem 11a-i? *

Copy and paste relevant sections from the manuscript (include quotes in quotation marks "like this" to indicate direct quotes from your manuscript), or elaborate on this item by providing additional information not in the ms, or briefly explain why the item is not applicable/relevant for your study

Not relevant, but it is specified in the text that this information can be found in the protocol: "For further information regarding masking and procedures, see the protocol (30)."
The protocol states: "The assessor will be blind to the type of treatment that will be administered to patients. In addition, this assessor will be different from the person who collects the measurements of the study results. As far as possible, GPs will also be blind to the intervention arm to which each patient is allocated, since their intervention should be based only on usual practice, based on the criteria set out in theGuide for the Treatment of Depression in Primary Care."

11a-ii) Discuss e.g., whether participants knew which intervention was the “intervention of interest” and which one was the “comparator”

Informed consent procedures (4a-ii) can create biases and certain expectations - discuss e.g., whether participants knew which intervention was the “intervention of interest” and which one was the “comparator”.

	1	2	3	4	5	
subitem not at all important	○	○	○	◉	○	essential

Borrar selección

Does your paper address subitem 11a-ii?

Copy and paste relevant sections from the manuscript (include quotes in quotation marks "like this" to indicate direct quotes from your manuscript), or elaborate on this item by providing additional information not in the ms, or briefly explain why the item is not applicable/relevant for your study

Yes: "The participants consented to their inclusion before finding out the treatment to which they were assigned and were permitted to withdraw from treatment at any time."

11b) If relevant, description of the similarity of interventions

(this item is usually not relevant for ehealth trials as it refers to similarity of a placebo or sham intervention to a active medication/intervention)

Does your paper address CONSORT subitem 11b? *

Copy and paste relevant sections from the manuscript (include quotes in quotation marks "like this" to indicate direct quotes from your manuscript), or elaborate on this item by providing additional information not in the ms, or briefly explain why the item is not applicable/relevant for your study

Not relevant, as the intervention to which the comparison is made (wait-list control or "iTAU") is the usual treatment applied by their GPs in PC.

12a) Statistical methods used to compare groups for primary and secondary outcomes

NPT: When applicable, details of whether and how the clustering by care providers or centers was addressed

Does your paper address CONSORT subitem 12a? *

Copy and paste relevant sections from the manuscript (include quotes in quotation marks "like this" to indicate direct quotes from your manuscript), or elaborate on this item by providing additional information not in the ms, or briefly explain why the item is not applicable/relevant for your study

Yes: "The primary analysis consisted of a comparison between the Intervention + iTAU and iTAU groups at three-month follow-up after the end of treatment, considering the main outcome as a continuous variable."..."The primary analysis was performed using an RM design on a modified intention-to-treat (ITT) basis – i.e., we analysed complete cases due to the high proportion of missingness and explored patterns of missing data. We used multilevel linear mixed regression models with the restricted maximum likelihood (RML) method for the estimation of parameters, controlling for age and sex as covariates. The 'treatment-by-time' interaction was calculated to determine possible differences between the study arms. The slope coefficient (B), representing the adjusted mean difference change, and its 95% confidence interval (95% CI), were calculated. Hedges' g, as an effect size measure of between-group differences, was calculated from the raw data – with g=0.2 (small effect), g=0.5 (intermediate effect), and g≥0.8 (large effect) (70). We used a two sided test with a .05 significance level.
The same analytical approach was used to perform secondary analyses for the main outcome at post-intervention, as well as for the components of the composite score, and for the secondary and mechanistic outcomes at post-intervention and at three-month follow-up. We corrected for multiple comparisons by adjusting the significance threshold based on the number of comparisons and the rank of the p-value according to the Benjamini-Hochberg procedure (71)."

12a-i) Imputation techniques to deal with attrition / missing values

Imputation techniques to deal with attrition / missing values: Not all participants will use the intervention/comparator as intended and attrition is typically high in ehealth trials. Specify how participants who did not use the application or dropped out from the trial were treated in the statistical analysis (a complete case analysis is strongly discouraged, and simple imputation techniques such as LOCF may also be problematic [4]).

	1	2	3	4	5	
subitem not at all important	○	○	○	○	●	essential

Borrar selección

Does your paper address subitem 12a-i? *

Copy and paste relevant sections from the manuscript (include quotes in quotation marks "like this" to indicate direct quotes from your manuscript), or elaborate on this item by providing additional information not in the ms, or briefly explain why the item is not applicable/relevant for your study

Yes: "The primary analysis was performed using an RM design on a modified intention-to treat (ITT) basis – i.e., we analysed complete cases due to the high proportion of missingness and explored patterns of missing data."
"Additional post-hoc sensitivity analyses were also conducted using Complier Average Causal Effect (CACE) / instrumental variable (IV) methods (72) to further investigate the impact of compliance with the programme on the composite (primary outcome) at postintervention and at three-month follow-up, while accounting for potential hidden confounding relationships. A participant in the Intervention + iTAU arm was considered a complier if he/she attended the two face-to-face sessions and also completed the six online modules. For this purpose, a two-stage least squares IV approach was employed. In the first stage regression, marital status, employment and diagnosis were included as predictors of compliance. In the second stage regression, age, sex and the composite at pre-intervention were introduced as predictors of the outcome at post-intervention or threemonth follow-up. The allocated group was used as an IV to define compliance. Results are presented in terms of unstandardized regression coefficients, along with their corresponding 95% CI and p-values."

12b) Methods for additional analyses, such as subgroup analyses and adjusted analyses

Does your paper address CONSORT subitem 12b? *

Copy and paste relevant sections from the manuscript (include quotes in quotation marks "like this" to indicate direct quotes from your manuscript), or elaborate on this item by providing additional information not in the ms, or briefly explain why the item is not applicable/relevant for your study

Yes: ""Additional post-hoc sensitivity analyses were also conducted using Complier Average Causal Effect (CACE) / instrumental variable (IV) methods (72) to further investigate the impact of compliance with the programme on the composite (primary outcome) at postintervention and at three-month follow-up, while accounting for potential hidden confounding relationships."...
"The role of positive affect, negative affect and openness to the future as mediators of improvements in the main outcome was explored. For this purpose, (a) the primary outcome at follow-up was considered the dependent variable; (b) pre-post differential scores of positive affect, negative affect and openness to the future were calculated and included as process variables; (c) the group condition (with two possibilities: Intervention + iTAU vs iTAU) was considered the independent variable. Models included the main outcome at pre-intervention, age and sex as covariates. The mediating analyses were conducted using path analyses for continuous dependent variables. Standardized regression coefficients of bootstrapped indirect effects were estimated, as well as their 95% CIs based on 10,000 bootstrapped samples, considering a significant mediating effect when the 95% CI did not include zero (73)."

X26) REB/IRB Approval and Ethical Considerations [recommended as subheading under "Methods"] (not a CONSORT item)

X26-i) Comment on ethics committee approval

	1	2	3	4	5	
subitem not at all important	○	○	○	◉	○	essential

Borrar selección

Does your paper address subitem X26-i?

Copy and paste relevant sections from the manuscript (include quotes in quotation marks "like this" to indicate direct quotes from your manuscript), or elaborate on this item by providing additional information not in the ms, or briefly explain why the item is not applicable/relevant for your study

Yes: "This study was approved by the research ethics committee in each of the autonomous communities involved (CEICA Aragon, CEIC Balearic Islands and the Regional Ethics and Research Committee of the province of Malaga) and was designed in accordance with the ethical standards laid down in the Declaration of Helsinki and its later amendments. Modifications to the published protocol (30) were approved by the research ethics committee of the autonomous community corresponding to the leading group (CEICA Aragon, PI16/0259)."

x26-ii) Outline informed consent procedures

Outline informed consent procedures e.g., if consent was obtained offline or online (how? Checkbox, etc.?), and what information was provided (see 4a-ii). See [6] for some items to be included in informed consent documents.

	1	2	3	4	5	
subitem not at all important	○	○	○	●	○	essential

Borrar selección

Does your paper address subitem X26-ii?

Copy and paste relevant sections from the manuscript (include quotes in quotation marks "like this" to indicate direct quotes from your manuscript), or elaborate on this item by providing additional information not in the ms, or briefly explain why the item is not applicable/relevant for your study

Yes, although explained in more detail in the protocol, it is specified that: "The inclusion criteria were as follows:... and (vii) willingness to participate in the study and signing the corresponding informed consent." "Patients were recruited by GPs working in PC centres of the previously mentioned Spanish autonomous communities, who subsequently sent the referral and consent forms of potential participants to the evaluating investigator" "The evaluating investigator then contacted the participants to schedule the screening assessments..." In Table 1. Overview of the program: "Face-to-face session 1 + M0. Programme presentation To present the online programme and train the patients in the procedure and to log in and use it on their home computers."

X26-iii) Safety and security procedures

Safety and security procedures, incl. privacy considerations, and any steps taken to reduce the likelihood or detection of harm (e.g., education and training, availability of a hotline)

	1	2	3	4	5	
subitem not at all important	○	○	○	◉	○	essential

Borrar selección

Does your paper address subitem X26-iii?

Copy and paste relevant sections from the manuscript (include quotes in quotation marks "like this" to indicate direct quotes from your manuscript), or elaborate on this item by providing additional information not in the ms, or briefly explain why the item is not applicable/relevant for your study

Yes: "In order to ensure the protection of personal information, Advanced Encryption Standard (AES) strategies regarding data encryption and use of personal passwords were implemented. The data were treated anonymously and confidentially and only used for the purposes of the study."
The programme consisted of: " two face-to-face individual sessions and six online individual and interactive therapeutic modules", as pointed out in the Table 1, in these sessions with their therapist they could: a) resolve doubts, b) carry out some of the most important practices, also taking into account the preferences of each patient, c) emphasise the continued practice of the strategies learned..."
In addition, they could leave the studio whenever they wished: "The participants ... were permitted to withdraw from treatment at any time."

RESULTS

13a) For each group, the numbers of participants who were randomly assigned, received intended treatment, and were analysed for the primary outcome

NPT: The number of care providers or centers performing the intervention in each group and the number of patients treated by each care provider in each center

Does your paper address CONSORT subitem 13a? *

Copy and paste relevant sections from the manuscript (include quotes in quotation marks "like this" to indicate direct quotes from your manuscript), or elaborate on this item by providing additional information not in the ms, or briefly explain why the item is not applicable/relevant for your study

Yes: "The primary analysis was performed using an RM design on a modified intention-to treat (ITT) basis – i.e., we analysed complete cases due to the high proportion of missingness and explored patterns of missing data." "As shown in Figure 1, after excluding 112 participants (who did not meet the inclusion criteria) from the initial 295, the remaining 183 individuals were randomly assigned to one of the two experimental conditions (Intervention + iTAU N = 93; iTAU N = 90)." "A total of 89 participants (95.7%) in the Intervention + iTAU arm attended the first face-to-face session, and 59 (63.4%) attended the second face-to-face session. The median (IQR) number of modules attended in the intervention arm was 4 (1, 7), with a mean (SD) of 4.02 (2.75). Post-intervention retention rates in the primary outcome (i.e., composite score) were 37.6% in the Intervention + iTAU arm and 54.4% in the iTAU arm, with rates of 49.5% and 55.6%, respectively, at follow-up."

13b) For each group, losses and exclusions after randomisation, together with reasons

Does your paper address CONSORT subitem 13b? (NOTE: Preferably, this is shown in a CONSORT flow diagram) *

Copy and paste relevant sections from the manuscript (include quotes in quotation marks "like this" to indicate direct quotes from your manuscript), or elaborate on this item by providing additional information not in the ms, or briefly explain why the item is not applicable/relevant for your study

Yes: "A total of 89 participants (95.7%) in the Intervention + iTAU arm attended the first face-to-face session, and 59 (63.4%) attended the second face-to-face session. The median (IQR) number of modules attended in the intervention arm was 4 (1, 7), with a mean (SD) of 4.02 (2.75). Post-intervention retention rates in the primary outcome (i.e., composite score) were 37.6% in the Intervention + iTAU arm and 54.4% in the iTAU arm, with rates of 49.5% and 55.6%, respectively, at follow-up." "At the three-month follow-up (primary endpoint), higher age, being separated/divorced and having a diabetes diagnosis (as well as taking antidiabetics and insulin) were significantly associated with a higher probability of attrition. On the other hand, being employed, engaging in household chores, being on sick leave and taking analgesics and antiepileptics were significantly associated with a lower probability of study attrition (Supplement 1)."
The reasons for dropping out/not completing the intervention or assessments are not discussed here as they are explored in the adherence and usability article.

13b-i) Attrition diagram

Strongly recommended: An attrition diagram (e.g., proportion of participants still logging in or using the intervention/comparator in each group plotted over time, similar to a survival curve) or other figures or tables demonstrating usage/dose/engagement.

	1	2	3	4	5	
subitem not at all important	○	○	○	◉	○	essential

Borrar selección

Does your paper address subitem 13b-i?

Copy and paste relevant sections from the manuscript or cite the figure number if applicable (include quotes in quotation marks "like this" to indicate direct quotes from your manuscript), or elaborate on this item by providing additional information not in the ms, or briefly explain why the item is not applicable/relevant for your study

The figure 1, shows Participant flow diagram (number of modules and fade to face sessions completed, and assessments). Other parameters, such as logging in, are not relevant here and are included in the adherence and usability article.

14a) Dates defining the periods of recruitment and follow-up

Does your paper address CONSORT subitem 14a? *

Copy and paste relevant sections from the manuscript (include quotes in quotation marks "like this" to indicate direct quotes from your manuscript), or elaborate on this item by providing additional information not in the ms, or briefly explain why the item is not applicable/relevant for your study

Yes, it is specified that: "The initial protocol originally stated (30) that four timepoints would be taken (i.e., baseline, post-treatment, three-month follow-up and six-month follow-up), with the last measurement being the primary endpoint. However, the outbreak of the COVID-19 pandemic in 2020 severely affected life in Spain." ..."As regards the present RCT, the pandemic outbreak coincided with the final phase of the study, immediately before the last follow-up measurement. Consequently, we had completed the initial baseline measurement, as well as delivery of the intervention, post-intervention measurement and the three-month follow-up measurement, with the six-month follow-up measurement yet to be conducted."

14a-i) Indicate if critical “secular events” fell into the study period

Indicate if critical “secular events” fell into the study period, e.g., significant changes in Internet resources available or “changes in computer hardware or Internet delivery resources”

	1	2	3	4	5	
subitem not at all important	○	○	○	◉	○	essential

Borrar selección

Does your paper address subitem 14a-i?

Copy and paste relevant sections from the manuscript (include quotes in quotation marks "like this" to indicate direct quotes from your manuscript), or elaborate on this item by providing additional information not in the ms, or briefly explain why the item is not applicable/relevant for your study

Yes: "However, the outbreak of the COVID-19 pandemic in 2020 severely affected life in Spain." ..."As regards the present RCT, the pandemic outbreak coincided with the final phase of the study, immediately before the last follow-up measurement. Consequently, we had completed the initial baseline measurement, as well as delivery of the intervention, post intervention measurement and the three-month follow-up measurement, with the six-month follow-up measurement yet to be conducted.Thus, the most significant impact of the pandemic was on data collection for the primary outcome at six months."..."Therefore, it was decided that the primary endpoint would be changed from six-month follow-up to three-month follow-up after treatment, which was a measure that remained unaffected by the complex and unusual circumstances brought about by the pandemic. Ultimately, the difficult situation at the time prevented us from performing the six-month follow-up measurement."

14b) Why the trial ended or was stopped (early)

Does your paper address CONSORT subitem 14b? *

Copy and paste relevant sections from the manuscript (include quotes in quotation marks "like this" to indicate direct quotes from your manuscript), or elaborate on this item by providing additional information not in the ms, or briefly explain why the item is not applicable/relevant for your study

Yes: "As a nationwide lockdown was declared in March 2020, this resulted in PC centres being closed to the public. Spanish PC centres, health care professionals, patients and the general population were all affected by the pandemic in a range of ways – emotionally, socially and, ultimately, in terms of the health services that were provided/received. As regards the present RCT, the pandemic outbreak coincided with the final phase of the study, immediately before the last follow-up measurement." ..."Thus, the most significant impact of the pandemic was on data collection for the primary outcome at six months. Several possibilities were considered, although they all pointed to the measurements being seriously compromised in terms of data collection and quality (e.g., uncertainty about when PC centres might reopen and social distancing issues). Consequently, the feasibility, validity and precision of the estimates – as well as the possibilities of generalizing the study results further than the specific circumstances affecting the Spanish population during the COVID-19 universal stressor pandemic – were brought into question."

15) A table showing baseline demographic and clinical characteristics for each group

NPT: When applicable, a description of care providers (case volume, qualification, expertise, etc.) and centers (volume) in each group

Does your paper address CONSORT subitem 15? *

Copy and paste relevant sections from the manuscript (include quotes in quotation marks "like this" to indicate direct quotes from your manuscript), or elaborate on this item by providing additional information not in the ms, or briefly explain why the item is not applicable/relevant for your study

Yes, Table 2. "Baseline characteristics of patients by treatment group" shows
Sociodemographic
data and Clinical data on each arm.

15-i) Report demographics associated with digital divide issues

In ehealth trials it is particularly important to report demographics associated with digital divide issues, such as age, education, gender, social-economic status, computer/Internet/ehealth literacy of the participants, if known.

	1	2	3	4	5	
subitem not at all important	○	○	○	○	◉	essential

Borrar selección

Does your paper address subitem 15-i? *

Copy and paste relevant sections from the manuscript (include quotes in quotation marks "like this" to indicate direct quotes from your manuscript), or elaborate on this item by providing additional information not in the ms, or briefly explain why the item is not applicable/relevant for your study

Yes, Table 2 reports demographics associated with digital divide issues, such as age, education, gender, social-economic status of the participants.

16) For each group, number of participants (denominator) included in each analysis and whether the analysis was by original assigned groups

16-i) Report multiple “denominators” and provide definitions

Report multiple “denominators” and provide definitions: Report N’s (and effect sizes) “across a range of study participation [and use] thresholds” [1], e.g., N exposed, N consented, N used more than x times, N used more than y weeks, N participants “used” the intervention/comparator at specific pre-defined time points of interest (in absolute and relative numbers per group). Always clearly define “use” of the intervention.

	1	2	3	4	5	
subitem not at all important	○	○	○	○	◉	essential

Borrar selección

Does your paper address subitem 16-i? *

Copy and paste relevant sections from the manuscript (include quotes in quotation marks "like this" to indicate direct quotes from your manuscript), or elaborate on this item by providing additional information not in the ms, or briefly explain why the item is not applicable/relevant for your study

Yes, on Table 3. "Descriptive statistics and main comparisons for primary and secondary outcomes at follow-up" the second column shows the n in each group analysed and the fifth column shows the effect sizes.

16-ii) Primary analysis should be intent-to-treat

Primary analysis should be intent-to-treat, secondary analyses could include comparing only “users”, with the appropriate caveats that this is no longer a randomized sample (see 18-i).

	1	2	3	4	5	
subitem not at all important	○	○	○	◉	○	essential

Borrar selección

Does your paper address subitem 16-ii?

Copy and paste relevant sections from the manuscript (include quotes in quotation marks "like this" to indicate direct quotes from your manuscript), or elaborate on this item by providing additional information not in the ms, or briefly explain why the item is not applicable/relevant for your study

As pointed out in the method (data analyses): "The primary analysis was performed using an RM design on a modified intention-to-treat (ITT) basis – i.e., we analysed complete cases due to the high proportion of missingness and explored patterns of missing data."

17a) For each primary and secondary outcome, results for each group, and the estimated effect size and its precision (such as 95% confidence interval)

Does your paper address CONSORT subitem 17a? *

Copy and paste relevant sections from the manuscript (include quotes in quotation marks "like this" to indicate direct quotes from your manuscript), or elaborate on this item by providing additional information not in the ms, or briefly explain why the item is not applicable/relevant for your study

Yes: "The study outcomes are reported by trial arm status at post-intervention (Supplement 2) and three-month follow-up (Table 3). At three-month follow-up (primary endpoint), there was evidence that the Intervention + iTAU group achieved a significantly greater reduction in the composite score (main outcome) compared to iTAU (B = −0.34; 95% CI = -0.64, -0.04), with small-to-medium effects (Hedges’ g = -0.39)."

17a-i) Presentation of process outcomes such as metrics of use and intensity of use

In addition to primary/secondary (clinical) outcomes, the presentation of process outcomes such as metrics of use and intensity of use (dose, exposure) and their operational definitions is critical. This does not only refer to metrics of attrition (13-b) (often a binary variable), but also to more continuous exposure metrics such as “average session length”. These must be accompanied by a technical description how a metric like a “session” is defined (e.g., timeout after idle time) [1] (report under item 6a).

	1	2	3	4	5	
subitem not at all important	○	○	○	●	○	essential

Borrar selección

Does your paper address subitem 17a-i?

Copy and paste relevant sections from the manuscript (include quotes in quotation marks "like this" to indicate direct quotes from your manuscript), or elaborate on this item by providing additional information not in the ms, or briefly explain why the item is not applicable/relevant for your study

Yes: "The results of the CACE/IV analyses represent an estimation of the intervention effect among the subpopulation of compliers in the intervention arm, compared to those in the control arm who would have complied with the intervention had they been offered it."
"Additional post-hoc sensitivity analyses were also conducted using Complier Average Causal Effect (CACE) / instrumental variable (IV) methods (72) to further investigate the impact of compliance with the programme on the composite (primary outcome) at post intervention and at three-month follow-up, while accounting for potential hidden confounding relationships. A participant in the Intervention + iTAU arm was considered a complier if he/she attended the two face-to-face sessions and also completed the six online modules. For this purpose, a two-stage least squares IV approach was employed. In the first stage regression, marital status, employment and diagnosis were included as predictors of compliance."
"The role of positive affect, negative affect and openness to the future as mediators of improvements in the main outcome was explored." ... "The mediating analyses were conducted using path analyses for continuous dependent variables. Standardized regression coefficients of bootstrapped indirect effects were estimated, as well as their 95% CIs based on 10,000 bootstrapped samples, considering a significant mediating effect when the 95% CI did not include zero (73)."

17b) For binary outcomes, presentation of both absolute and relative effect sizes is recommended

Does your paper address CONSORT subitem 17b? *

Copy and paste relevant sections from the manuscript (include quotes in quotation marks "like this" to indicate direct quotes from your manuscript), or elaborate on this item by providing additional information not in the ms, or briefly explain why the item is not applicable/relevant for your study

Not applicable, no binary outcomes reported. Only absolute effects sizes reported: "with small-to-large effects (Hedges' g ranging from 0.36 to 1.13 in absolute value)" ..."with small-to-large effects (Hedges' g ranging from 0.38 to 0.88 in absolute value)."

18) Results of any other analyses performed, including subgroup analyses and adjusted analyses, distinguishing pre-specified from exploratory

Does your paper address CONSORT subitem 18? *

Copy and paste relevant sections from the manuscript (include quotes in quotation marks "like this" to indicate direct quotes from your manuscript), or elaborate on this item by providing additional information not in the ms, or briefly explain why the item is not applicable/relevant for your study

Yes: "At the three-month follow-up (primary endpoint), higher age, being separated/divorced and having a diabetes diagnosis (as well as taking antidiabetics and insulin) were significantly associated with a higher probability of attrition. On the other hand, being employed, engaging in household chores, being on sick leave and taking analgesics and antiepileptics were significantly associated with a lower probability of study attrition (Supplement 1)."

18-i) Subgroup analysis of comparing only users

A subgroup analysis of comparing only users is not uncommon in ehealth trials, but if done, it must be stressed that this is a self-selected sample and no longer an unbiased sample from a randomized trial (see 16-iii).

	1	2	3	4	5	
subitem not at all important	○	○	○	◉	○	essential

Borrar selección

Does your paper address subitem 18-i?

Copy and paste relevant sections from the manuscript (include quotes in quotation marks "like this" to indicate direct quotes from your manuscript), or elaborate on this item by providing additional information not in the ms, or briefly explain why the item is not applicable/relevant for your study

"The primary analysis was performed using an RM design on a modified intention-to-treat (ITT) basis – i.e., we analysed complete cases due to the high proportion of missingness and explored patterns of missing data."... "The same analytical approach was used to perform secondary analyses for the main outcome at post-intervention, as well as for the components of the composite score, and for the secondary and mechanistic outcomes at post-intervention and at three-month follow-up. We corrected for multiple comparisons by adjusting the significance threshold based on the number of comparisons and the rank of the p-value according to the Benjamini-Hochberg procedure (71)."

19) All important harms or unintended effects in each group

(for specific guidance see CONSORT for harms)

Does your paper address CONSORT subitem 19? *

Copy and paste relevant sections from the manuscript (include quotes in quotation marks "like this" to indicate direct quotes from your manuscript), or elaborate on this item by providing additional information not in the ms, or briefly explain why the item is not applicable/relevant for your study

No harms or unintended effects occurred.

19-i) Include privacy breaches, technical problems

Include privacy breaches, technical problems. This does not only include physical "harm" to participants, but also incidents such as perceived or real privacy breaches [1], technical problems, and other unexpected/unintended incidents. "Unintended effects" also includes unintended positive effects [2].

	1	2	3	4	5	
subitem not at all important	○	○	○	◉	○	essential

Borrar selección

Does your paper address subitem 19-i?

Copy and paste relevant sections from the manuscript (include quotes in quotation marks "like this" to indicate direct quotes from your manuscript), or elaborate on this item by providing additional information not in the ms, or briefly explain why the item is not applicable/relevant for your study

Not applicable, no privacy breaches, technical problems, or other unexpected/unintended incidents. If participants reported perceived privacy breaches or technical problems, these will be analysed and reported in other paper (from the qualitative or adherence/usability study).

19-ii) Include qualitative feedback from participants or observations from staff/researchers

Include qualitative feedback from participants or observations from staff/researchers, if available, on strengths and shortcomings of the application, especially if they point to unintended/unexpected effects or uses. This includes (if available) reasons for why people did or did not use the application as intended by the developers.

	1	2	3	4	5	
subitem not at all important	○	○	○	●	○	essential

Borrar selección

Does your paper address subitem 19-ii?

Copy and paste relevant sections from the manuscript (include quotes in quotation marks "like this" to indicate direct quotes from your manuscript), or elaborate on this item by providing additional information not in the ms, or briefly explain why the item is not applicable/relevant for your study

Not shown in this article, as these issues were recorded in the qualitative study or adherence/usability study (barriers and facilitators, opinions, expectations,...).

DISCUSSION

22) Interpretation consistent with results, balancing benefits and harms, and considering other relevant evidence

NPT: In addition, take into account the choice of the comparator, lack of or partial blinding, and unequal expertise of care providers or centers in each group

22-i) Restate study questions and summarize the answers suggested by the data, starting with primary outcomes and process outcomes (use)

Restate study questions and summarize the answers suggested by the data, starting with primary outcomes and process outcomes (use).

	1	2	3	4	5	
subitem not at all important	○	○	○	○	◉	essential

Borrar selección

Does your paper address subitem 22-i? *

Copy and paste relevant sections from the manuscript (include quotes in quotation marks "like this" to indicate direct quotes from your manuscript), or elaborate on this item by providing additional information not in the ms, or briefly explain why the item is not applicable/relevant for your study

Yes: "The main aim of the present trial was to evaluate the effectiveness of a low-intensity psychotherapy programme (Intervention + iTAU) applied in a hybrid form, with face-to-face and internet-based sessions (i.e., blended), for the treatment of multimorbidity between mild-to-moderate severity depression and either type 2 diabetes or chronic low back pain in PC, compared to a group that only received iTAU. It was observed that the Intervention + iTAU group achieved a significantly greater reduction in the composite score (main outcome) compared to iTAU, with large effects at post-intervention and small-to-medium effects at follow-up. In addition, compared to iTAU, the Intervention + iTAU group showed greater reductions in depressive symptomatology, low back pain disability and negative affect at post-intervention, although not at follow-up, where only reductions in depression and negative affect were maintained. On the other hand, greater improvements in perceived health, positive affect and openness to the future were observed in the Intervention + iTAU group vs iTAU, with small-to-large effects at both timepoints. However, no significant differences in glycosylated haemoglobin or pain intensity were identified in the comparison between the Intervention + iTAU group and the iTAU, either at post-intervention or at three month follow-up."

22-ii) Highlight unanswered new questions, suggest future research

Highlight unanswered new questions, suggest future research.

	1	2	3	4	5	
subitem not at all important	○	○	○	◉	○	essential

Borrar selección

Does your paper address subitem 22-ii?

Copy and paste relevant sections from the manuscript (include quotes in quotation marks "like this" to indicate direct quotes from your manuscript), or elaborate on this item by providing additional information not in the ms, or briefly explain why the item is not applicable/relevant for your study

Yes: "It is possible that pain intensity, being a variable that is difficult to modify, exhibits effects that are lost earlier, and this was evident in both cases, although the loss occurred in the medium term. As far as we know, there are no studies applying blended psychotherapy in patients with chronic pain and depression. Therefore, reaching a solid conclusion in this regard is challenging, highlighting the need for further studies in this field."
"Thus, the method of implementing the intervention could play a decisive role. Given that our intervention was blended, such effects might have been mitigated. This could also be associated with the small sample size (resulting in reduced statistical power) achieved when recruiting patients with diabetes, along with the limited follow-up time (three months). The HbA1c variable represents the proportion of haemoglobin in the blood that has bound to glucose over an extended period. Therefore, a more extended follow-up time may be necessary to observe significant results, as demonstrated by Hoyo et al. (104) where HbA1c levels continued to decrease at 12- and 18-month follow-ups."
"Our results underscore the crucial role of affect, both positive and negative, as potential mediators in the functioning of the intervention, influencing improvements in the composite main outcome. Understanding how affective states mediate the impact of the intervention is pivotal for tailoring and optimizing future treatment developments. This finding emphasizes the need for interventions that consider the affective dimension, not only addressing the negative valence depressive symptoms, but also enhancing positive emotional experiences. While our study contributes to this understanding, it is noteworthy that similar results were found when using MBCT to reduce the risk of relapse/recurrence in major depressive disorder (109)."
"There is also a proposal that patients with diabetes and mild depression might perceive low mood as a feature of their diabetes rather than a separate condition to be treated, potentially influencing treatment adherence and completion (111). As suggested by other authors (29), further exploration of the conceptualization of depression in type 2 diabetes and its impact on programme uptake and the benefit of treatment is recommended." ... "We encourage further research to validate the results reached in this study, including the exploration of subgroups that could not be adequately examined due to the discussed limitations. Additionally, we advocate an exploration of the conceptualization of depression in type 2 diabetes for the purpose of shedding light on its effects on adherence indicators, acceptance and the efficacy of psychological interventions in these patients. Analysing the implementation of such interventions in routine clinical practice is also warranted."

20) Trial limitations, addressing sources of potential bias, imprecision, and, if relevant, multiplicity of analyses

20-i) Typical limitations in ehealth trials

Typical limitations in ehealth trials: Participants in ehealth trials are rarely blinded. Ehealth trials often look at a multiplicity of outcomes, increasing risk for a Type I error. Discuss biases due to non-use of the intervention/usability issues, biases through informed consent procedures, unexpected events.

	1	2	3	4	5	
subitem not at all important	○	○	○	○	◉	essential

Borrar selección

Does your paper address subitem 20-i? *

Copy and paste relevant sections from the manuscript (include quotes in quotation marks "like this" to indicate direct quotes from your manuscript), or elaborate on this item by providing additional information not in the ms, or briefly explain why the item is not applicable/relevant for your study

Yes: "However, the study has significant limitations. A notable challenge was the high attrition rate, which impacted the study's statistical power. Recruitment and retention difficulties in randomized controlled trials targeting comorbid physical and mental illnesses have been documented in prior studies (110), and specific details of the present study will be discussed in a forthcoming publication focused on implementation. Nevertheless, it is important to highlight that, in line with the earlier recruitment challenges, dropouts were more prevalent among older patients and those with type 2 diabetes. This trend might be attributed to challenges in managing the use of Internet and e-mail, and acquisition of the basic computer skills required for active participation. Similar observations were made in a study by Clarke et al. (29) focusing on patients with type 2 diabetes and depression. This study reported higher attrition and mean age compared to other ICT-delivered interventions in chronic low back pain and depression (88,91) and diabetes and depression (79,80). It is noteworthy that the latter studies also included type 1 diabetes, contributing to differences in the age distribution of participants. There is also a proposal that patients with diabetes and mild depression might perceive low mood as a feature of their diabetes rather than a separate condition to be treated, potentially influencing treatment adherence and completion (111). As suggested by other authors (29), further exploration of the conceptualization of depression in type 2 diabetes and its impact on programme uptake and the benefit of treatment is recommended. Additionally, like many clinical trials, our study faced disruptions due to the COVID-19 pandemic (112), preventing the implementation of necessary follow-up measures, particularly for patients with diabetes. Given their heightened vulnerability to the virus, this situation led to the loss of valuable follow-up data. Finally, we observed a varying proportion of losses between the intervention and control groups post-intervention, although this effect disappeared at the subsequent primary endpoint. Similar effects were noted in prior studies (29,79,80,91,101) and could be attributed to some extent to the control groups being wait-listed, leading participants to initially complete the assessment and refrain from dropping out in anticipation of receiving the intervention at a later point."

21) Generalisability (external validity, applicability) of the trial findings

NPT: External validity of the trial findings according to the intervention, comparators, patients, and care providers or centers involved in the trial

21-i) Generalizability to other populations

Generalizability to other populations: In particular, discuss generalizability to a general Internet population, outside of a RCT setting, and general patient population, including applicability of the study results for other organizations

	1	2	3	4	5	
subitem not at all important	○	○	○	◉	○	essential

Borrar selección

Does your paper address subitem 21-i?

Copy and paste relevant sections from the manuscript (include quotes in quotation marks "like this" to indicate direct quotes from your manuscript), or elaborate on this item by providing additional information not in the ms, or briefly explain why the item is not applicable/relevant for your study

Yes: "As far as we know, there are no studies applying blended psychotherapy in patients with chronic pain and depression. Therefore, reaching a solid conclusion in this regard is challenging, highlighting the need for further studies in this field."
"Specifically, most type 2 diabetes patients were found to be elderly and did not have access to the Internet or e-mail, nor did they have the basic computer skills that would allow them to participate in the study. This considerably slowed down the recruitment phase of the trial."

21-ii) Discuss if there were elements in the RCT that would be different in a routine application setting

Discuss if there were elements in the RCT that would be different in a routine application setting (e.g., prompts/reminders, more human involvement, training sessions or other co-interventions) and what impact the omission of these elements could have on use, adoption, or outcomes if the intervention is applied outside of a RCT setting.

	1	2	3	4	5	
subitem not at all important	○	○	○	◉	○	essential

Borrar selección

Does your paper address subitem 21-ii?

Copy and paste relevant sections from the manuscript (include quotes in quotation marks "like this" to indicate direct quotes from your manuscript), or elaborate on this item by providing additional information not in the ms, or briefly explain why the item is not applicable/relevant for your study

Yes: "Analysing the implementation of such interventions in routine clinical practice is also warranted."

OTHER INFORMATION

23) Registration number and name of trial registry

Does your paper address CONSORT subitem 23? *

Copy and paste relevant sections from the manuscript (include quotes in quotation marks "like this" to indicate direct quotes from your manuscript), or elaborate on this item by providing additional information not in the ms, or briefly explain why the item is not applicable/relevant for your study

Yes: "Trial registration: ClinicalTrials.gov NCT03426709. Registered on 8 February 2018."

24) Where the full trial protocol can be accessed, if available

Does your paper address CONSORT subitem 24? *

Cite a Multimedia Appendix, other reference, or copy and paste relevant sections from the manuscript (include quotes in quotation marks "like this" to indicate direct quotes from your manuscript), or elaborate on this item by providing additional information not in the ms, or briefly explain why the item is not applicable/relevant for your study

Yes: "Three major changes were made to the original protocol (30),...", "For further information regarding masking and procedures, see the protocol (30)."

25) Sources of funding and other support (such as supply of drugs), role of funders

Does your paper address CONSORT subitem 25? *

Copy and paste relevant sections from the manuscript (include quotes in quotation marks "like this" to indicate direct quotes from your manuscript), or elaborate on this item by providing additional information not in the ms, or briefly explain why the item is not applicable/relevant for your study

Yes: "This research was financed by a FIS grant from the Carlos III Health Institute of the Spanish Ministry of Economy and Competitiveness (PI16/00962). The first author had a FI predoctoral contract awarded by the Carlos III Health Institute (ISCIII; FI17/00180) to the Institute of Health Research of Aragon (IIS), Zaragoza, in order to conduct this study. The funding body had no role in the design of the study and data collection, nor did it play any part in the analysis, interpretation of data or the writing of manuscripts. The funding entity audited the trial execution."

X27) Conflicts of Interest (not a CONSORT item)

X27-i) State the relation of the study team towards the system being evaluated

In addition to the usual declaration of interests (financial or otherwise), also state the relation of the study team towards the system being evaluated, i.e., state if the authors/evaluators are distinct from or identical with the developers/sponsors of the intervention.

	1	2	3	4	5	
subitem not at all important	○	○	○	◉	○	essential

Borrar selección

Does your paper address subitem X27-i?

Copy and paste relevant sections from the manuscript (include quotes in quotation marks "like this" to indicate direct quotes from your manuscript), or elaborate on this item by providing additional information not in the ms, or briefly explain why the item is not applicable/relevant for your study

Yes: "Institute of Health Research of Aragon (IIS), Zaragoza, in order to conduct this study. The funding body had no role in the design of the study and data collection, nor did it play any part in the analysis, interpretation of data or the writing of manuscripts."
"An independent researcher unrelated to the research team generated the individual randomization list using a randomization software. A researcher from the research team not involved in any other project-related task, together with an independent general practitioner, performed data monitoring tasks."

About the CONSORT EHEALTH checklist

As a result of using this checklist, did you make changes in your manuscript? *

- ○ yes, major changes
- ○ yes, minor changes
- ◉ no

What were the most important changes you made as a result of using this checklist?

Tu respuesta

How much time did you spend on going through the checklist INCLUDING making changes in your manuscript *

It took me approximately 6 hours.

As a result of using this checklist, do you think your manuscript has improved? *

- (x) yes
- ( ) no
- ( ) Otro:

Would you like to become involved in the CONSORT EHEALTH group?

This would involve for example becoming involved in participating in a workshop and writing an "Explanation and Elaboration" document

- ( ) yes
- (x) no
- ( ) Otro:

Borrar selección

Any other comments or questions on CONSORT EHEALTH

Tu respuesta

STOP - Save this form as PDF before you click submit
To generate a record that you filled in this form, we recommend to generate a PDF of this page (on a Mac, simply select "print" and then select "print as PDF") before you submit it.

When you submit your (revised) paper to JMIR, please upload the PDF as supplementary file.

Don't worry if some text in the textboxes is cut off, as we still have the complete information in our database. Thank you!

Final step: Click submit !
Click submit so we have your answers in our database!

Enviar

Borrar formulario

Nunca envíes contraseñas a través de Formularios de Google.

Este contenido no ha sido creado ni aprobado por Google. Notificar uso inadecuado - Términos del Servicio - Política de Privacidad

Google Formularios
